# TCTP and CSN4 control cell cycle progression and development by regulating CULLIN1 neddylation in plants and animals

**DOI:** 10.1371/journal.pgen.1007899

**Published:** 2019-01-29

**Authors:** Léo Betsch, Véronique Boltz, Florian Brioudes, Garance Pontier, Victor Girard, Julie Savarin, Barbara Wipperman, Pierre Chambrier, Nicolas Tissot, Moussa Benhamed, Bertrand Mollereau, Cécile Raynaud, Mohammed Bendahmane, Judit Szécsi

**Affiliations:** 1 Laboratoire Reproduction et Développement des Plantes, Univ Lyon, ENS de Lyon, UCB Lyon 1, CNRS, INRA, UMS 3444 Biosciences Lyon Gerland, Ecole Normale Supérieure, Lyon, France; 2 Laboratory of Biology and Modelling of the Cell, UMR5239 CNRS/ENS de Lyon, INSERM U1210, UMS 3444 Biosciences Lyon Gerland, Univ Lyon, Lyon, France; 3 Institute of Plant Sciences Paris-Saclay (IPS2), CNRS, INRA, University Paris-Sud, University of Evry, University Paris-Diderot, Sorbonne Paris-Cite, University of Paris-Saclay, Orsay, France; Cornell University, UNITED STATES

## Abstract

Translationally Controlled Tumor Protein (TCTP) controls growth by regulating the G1/S transition during cell cycle progression. Our genetic interaction studies show that TCTP fulfills this role by interacting with CSN4, a subunit of the COP9 Signalosome complex, known to influence CULLIN-RING ubiquitin ligases activity by controlling CULLIN (CUL) neddylation status. In agreement with these data, downregulation of *CSN4* in *Arabidopsis* and in tobacco cells leads to delayed G1/S transition comparable to that observed when *TCTP* is downregulated. Loss-of-function of *AtTCTP* leads to increased fraction of deneddylated CUL1, suggesting that AtTCTP interferes negatively with COP9 function. Similar defects in cell proliferation and CUL1 neddylation status were observed in *Drosophila* knockdown for *dCSN4* or *dTCTP*, respectively, demonstrating a conserved mechanism between plants and animals. Together, our data show that CSN4 is the missing factor linking TCTP to the control of cell cycle progression and cell proliferation during organ development and open perspectives towards understanding TCTP’s role in organ development and disorders associated with TCTP miss-expression.

## Author summary

During organism development, the correct implementation of organs with unique shape, size and function, is the result of coordinated cellular processes, such as cell proliferation and expansion. Deregulation of these processes affect human health and can lead to severe diseases. While plants and animals have largely diverged in several aspects, some biological functions, such as cell proliferation, are conserved between these kingdoms. Previously we reported that the Translationally Controlled Tumor Protein (TCTP), a highly-conserved protein among all eukaryotes, positively regulates cell proliferation and this role is conserved between plants and animals. In agreement with these data, animals TCTP was reported to highly accumulate in tumor cells, and thus represents a target for cancer research and therapies. To discover how TCTP regulates cell proliferation, we conducted studies to identify factors acting in the TCTP pathway. Using the model plant Arabidopsis, we identified that TCTP fulfil its role by interacting with CSN4, a subunit of the conserved COP9 complex. TCTP interferes with the role of COP9 to regulate the downstream complex CRL known to control cell proliferation in eukaryotes. We further demonstrate that this role is conserved in the fly Drosophila, thus corroborating the conservation of TCTP pathway between plants and animals. We believe that, the data here will provide exciting perspectives, beyond plant research, that will help understand developmental disorders associated with TCTP misfunction, such as cancer.

## Introduction

The correct implementation of organs with unique shape, size and function is fundamental to the development of all multicellular organisms and is the result of coordinated cellular processes requiring key molecular actors. One such key player in eukaryotes is the Translationally Controlled Tumor Protein (TCTP). TCTP was discovered in the 1980’s as a protein positively regulated at the translational level in many tumors [1,2]. TCTP is a highly-conserved protein found in all eukaryotes. TCTP was reported to be involved in several cellular processes, including cell proliferation, cell growth, malignant transformation, apoptosis and protection against various cellular stresses [3–8].

In plants as in animals, TCTP loss of function leads to embryonic lethality, because of slower cell cycle progression and reduced cell proliferation [6] and reduced cell proliferation associated with excessive cell death [9,10], respectively. The fact that TCTP loss of function is lethal demonstrates its major role in eukaryote development, but also hampered the full comprehension of its exact roles. By performing embryo rescue, we generated the first *TCTP* full knockout adult organism which allowed us to demonstrate that TCTP controls cell cycle progression by regulating G1/S transition and that this role is conserved between plants and animals [6]. However, how TCTP controls the G1/S transition and cell proliferation remains unknown in plants as in animals. To better understand such a role, we searched for TCTP interacting proteins and identified that TCTP interacts physically with CSN4, one of the eight subunits of the Constitutive Photomorphogenesis 9 (COP9) Signalosome (CSN), initially discovered in plants [11,12] and conserved in eukaryotes [13]. CSN regulates the activity, the assembly and/or subunits stability of CULLIN-RING ubiquitin Ligase (CRLs) complexes, a major class of the E3 ligase complexes in eukaryotes [14] involved in the polyubiquitination of proteins targeted to degradation [15]. The proper functioning of CRL has been shown to be absolutely required for various functions associated with the control of cell cycle, transcription, stress response, self-incompatibility, pathogen defense and hormones and light signaling [16–18].

CSN mainly regulates CLR activity through the removal of the post-translational modification RUB/NEDD8 (Related to UBiquitin/Neural Precursor Cell Expressed, Developmentally Down-Regulated 8) from its CULLIN (CUL) subunit [18]. The eight subunits of CSN are mandatory for the deneddylation activity [19–21].

Here, we show that TCTP interacts physically and genetically with CSN4 to control cell cycle progression. Our data demonstrate that downregulation of *TCTP* or *CSN4* both leads to retarded G1/S transition, slower cell cycle progression and delay in plant development. Consistent with these data, knockout of TCTP is associated with increased CUL1 deneddylation. Conversely, over-accumulation of TCTP leads to accelerated cell cycle and plant development, associated with over-accumulation of neddylated CUL1. We also show that in *Drosophila*, the downregulation of dTCTP or dCSN4 is associated with defects in cell proliferation. Moreover, similar to our observations in plants, dTCTP downregulation is accompanied by increased CUL1 deneddylation. These data suggest that TCTP interacts with CSN4 and such interaction acts on the CUL1 neddylation status and affects CRL complex activity early during cell cycle progression influencing organ development both in plants and animals.

## Results

### TCTP and CSN4 interact physically and genetically to control growth

To gain insights into how TCTP controls cell cycle progression, we searched for its interacting proteins. Immunoprecipitation followed by mass spectrometry (IP/MS) experiments were performed using protein extracts from *Arabidopsis* line expressing *35S*::*AtTCTP-GFP*. Wild-type (WT) Col-0 and free-GFP-overexpressing plants lines (*35S*::*GFP*) served as controls. Among the proteins that co-immunoprecipitated with TCTP-GFP, but were absent in the control samples, we identified AtCSN4, a subunit of COP9 Signalosome, that was previously shown to be involved in the regulation of cell cycle progression [22]. To confirm this interaction, we generated line *AtTCTPg-GFP* that expresses a TCTP-GFP fusion under the control of its own promoter and line *AtTCTPg-GFP/35S*::*AtCSN4-Flag*, that, in addition, expresses *AtCSN4*-Flag under the control of the constitutive CaMV 35S promoter (*35S*::*AtCSN4-Flag*). Anti-GFP antibody was used to immunoprecipitate the TCTP-GFP fraction from total proteins extracted from 10 days-old seedlings (S1A Fig), or from mature green seeds harvested from green siliques (S1B Fig).

The presence of CSN4 (46 kDa) was then revealed in the immunoprecipitated fraction. AtCSN4 was found in the AtTCTP-GFP enriched fraction from both 10 days-old seedlings and mature green seeds (S1A and S1B Fig, respectively). No trace of AtCSN4 was detected in WT Col-0 used as control and treated in parallel.

**Fig 1:**
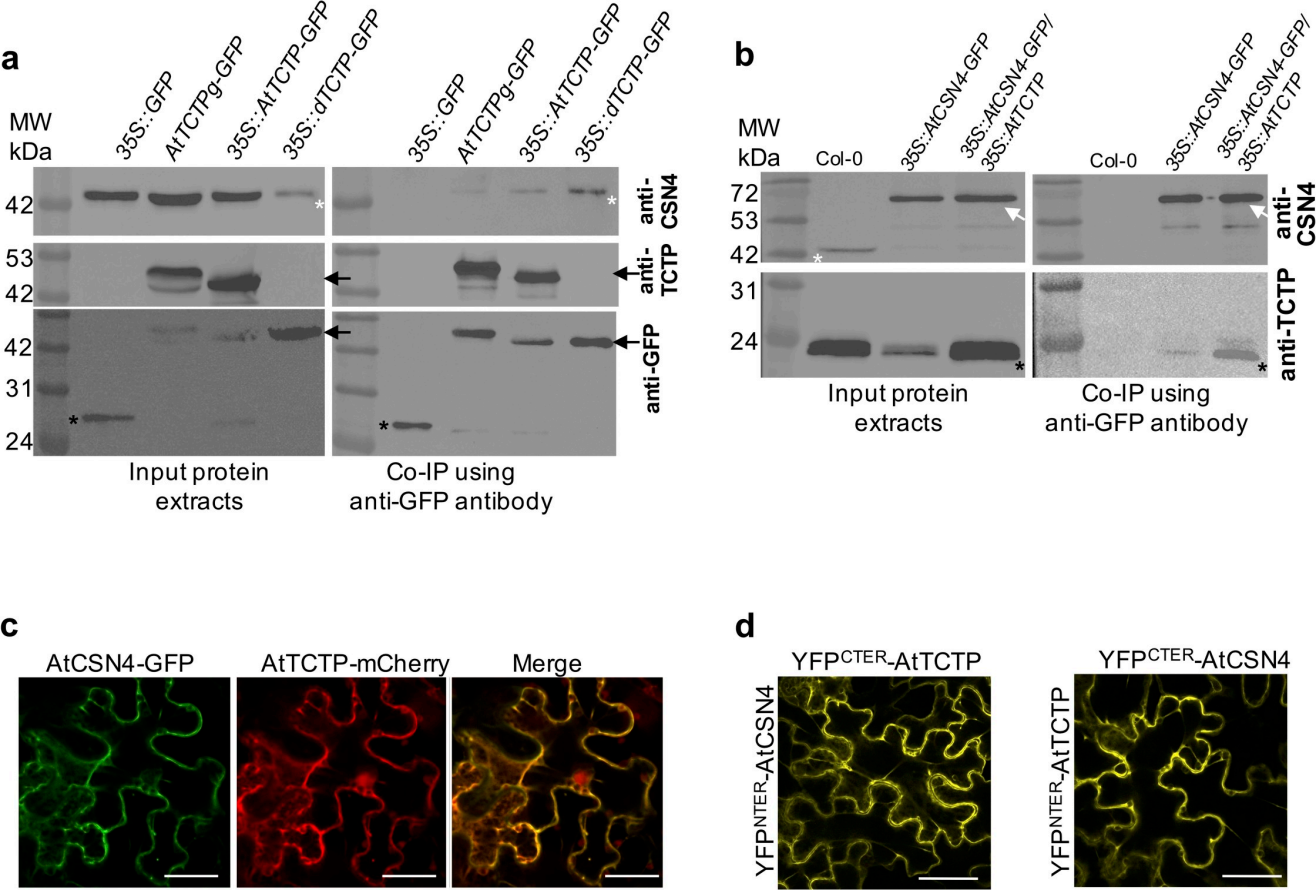
AtTCTP and AtCSN4 interact *in vitro* and *in vivo*. **(a)** TCTP interacting proteins were co-immunoprecipitated from protein extracts prepared from inflorescences of *35S*::*GFP*, *AtTCTPg-GFP*, *35S*::*AtTCTP-GFP* and *35S*::*dTCTP-GFP*/*tctp* plants using anti-GFP coupled magnetic beads. Co-immunoprecipitated proteins were detected by Western blotting using anti-CSN4 (upper panel), anti-TCTP (middle panel) or anti-GFP (lower panel) antibodies. White asterisks: CSN4 protein; black arrows: TCTP-GFP protein; black asterisks: free GFP. **(b)** CSN4 interacting proteins were co-immunoprecipitated from protein extracts prepared from inflorescences of Col-0, *35S*::*AtCSN4-GFP* and *35S*::*AtCSN4-GFP/35S*::*AtTCTP* plants using anti-GFP coupled magnetic beads. Co-immunoprecipitated proteins were detected by Western blotting using anti-CSN4 (upper panel) or anti-TCTP (lower panel) antibodies. White asterisks: CSN4 protein; white arrow: CSN4-GFP protein; black asterisks: TCTP protein. (**c**) AtCSN4-GFP (green) and AtTCTP-mCherry (red) co-localize in tobacco leaves. Bars: 50 μm. (**d**) Bimolecular Fluorescence complementation experiments in tobacco leaves show that AtTCTP and AtCSN4 fused with either C- or N-terminal YFP moieties, interact *in vivo*. Bars: 50 μm. As shown in S2 Fig, no signal was observed in the control BiFC assays in which AtTCTP or AtCSN4 fused with N- or C-terminal YFP moieties was co-infiltrated with an empty plasmid.

Next, we confirmed that endogenous AtCSN4 is able to co-immunoprecipitate with AtTCTP. For this, we performed immunoprecipitation on total proteins extracted from inflorescence of *AtTCTPg-GFP* and *35S*::*AtTCTP-GFP*. As shown in Fig 1A, CSN4 co-immunoprecipitated with AtTCTP-GFP in both lines (Figs 1A and S1C). The interaction was further confirmed by pulling-down AtCSN4. Using plant lines *35S*::*AtCSN4-GFP*, overexpressing AtCSN4, and *35S*::*AtCSN4-GFP/35S*::*AtTCTP*, overexpressing both proteins, we show that TCTP co-immunoprecipitates with AtCSN4-GFP from both plant lines (Figs 1B and S1D).

Finally, to address if *Drosophila* TCTP also interacts with CSN4, we performed co-immunoprecipitation experiments using plant lines expressing *Drosophila dTCTP* in *tctp* knockout genetic background (line *35S*::*dTCTP-GFP*). Previously we demonstrated that dTCTP was able to fully complement loss-of-function *tctp* [6]. As shown in Fig 1A, CSN4 co-immunoprecipitated with AtTCTP-GFP and also with dTCTP-GFP (Figs 1A and S1C), thus suggesting conservation of the TCTP/CSN4 interaction between plants and animals.

Next, we investigated the sub-cellular localization of CSN4 compared to TCTP. In agreement with co-immunoprecipitation data, co-expression of AtTCTP-mCherry and AtCSN4-GFP in tobacco leaf epidermal cells showed that these proteins co-localize *in planta* (Fig 1C). To further investigate AtTCTP-AtCSN4 interaction, we performed Bi-molecular Fluorescence Complementation (BiFC [23]) experiments by protein fusion to split YFP moieties and co-expression in tobacco cells. The data demonstrate that AtTCTP and AtCSN4 interact *in planta* (Fig 1D), confirming the co-immunoprecipitation results. Similar BiFC complementation results were obtained regardless if AtTCTP and AtCSN4 were fused to YFP^Cter^ or YFP^Nter^ (Fig 1D). BiFC experiments also showed that both AtTCTP and AtCSN4 were able to form homodimers (S2 Fig). However, the localization of AtTCTP-AtCSN4 heterodimer was distinct from that of the AtTCTP or AtCSN4 homodimers, thus corroborating TCTP-CSN4 *in vivo* interaction. No signal was observed when one of the vectors was empty (S2 Fig). Taken together these data demonstrate that AtTCTP and AtCSN4 interact physically *in vivo*.

To understand the biological significance of the AtTCTP-AtCSN4 interaction, we performed genetic interaction analyses using knockdown or overexpressor lines for both proteins. Previously, we showed that while *AtTCTP* knockout leads to embryo lethality, its down-regulation *via* RNAi (*RNAi-AtTCTP* lines) gave viable plants that nevertheless showed developmental defects [6]. Similar to plants mutants for *AtTCTP*, plants knockout for *AtCSN4* showed severe developmental defects during early seed germination leading to seedling death in few days after germination (S3 Fig), thus in agreement with Dohmann *et al*. [22]. To overcome this difficulty, we generated knockdown plants *via* expression of a RNAi directed against *AtCSN4* (line *RNAi-AtCSN4*). Western blot analyses confirmed the downregulation of *AtTCTP* and *AtCSN4* in the corresponding RNAi lines (S4 Fig).

**Fig 2:**
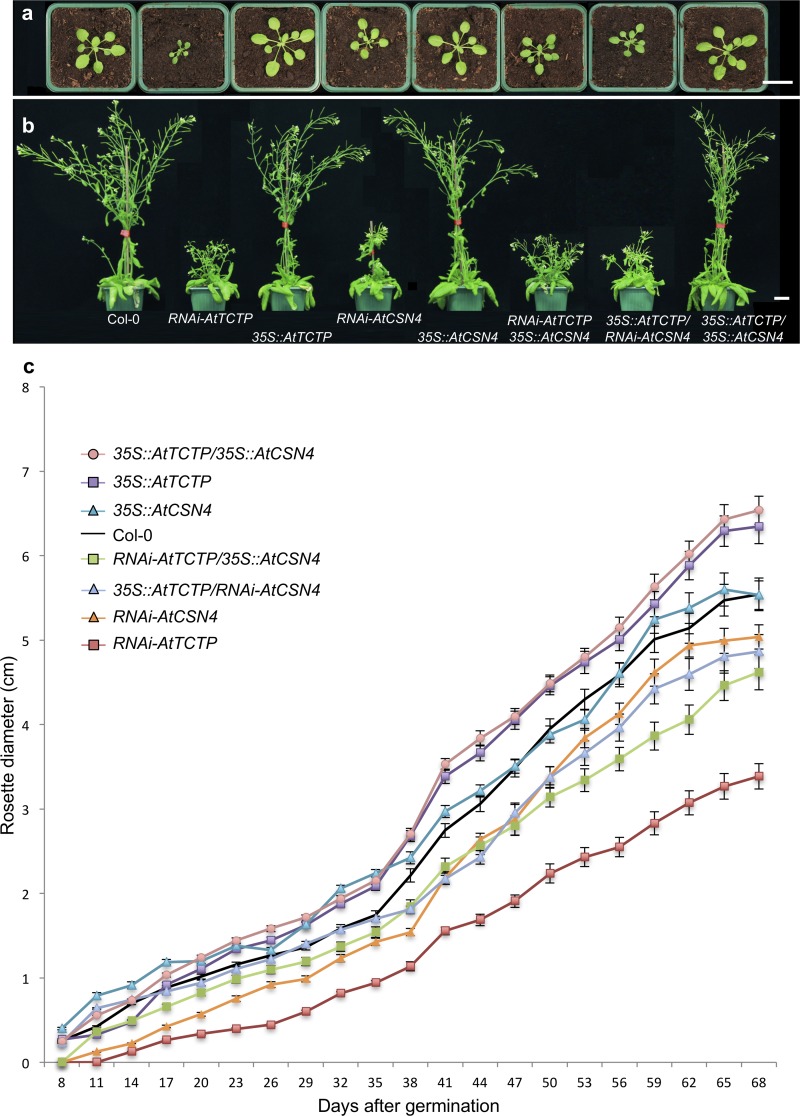
AtTCTP and AtCSN4 control plant development. **(a-b)** Plants knockdown for *CSN4* (*RNAi-AtCSN4*) exhibit a delay in development similar to that of *RNAi-AtTCTP* plants. *RNAi-AtCSN4* or *RNAi-AtTCTP* plants overexpressing *AtTCTP* or *AtCSN4*, respectively, show similar phenotype as the simple RNAi lines. Pictures of plants were taken 59 days **(a**) and 92 days **(b)** after sowing. Bars: 2 cm. (**c**) Rosette diameter was measured from 8 days until 68 days after germination. The error bars represent standard errors. n = 18.

*RNAi-AtCSN4* exhibited significant delay in rosette development compared to Col-0, thus a similar phenotype as *RNAi-AtTCTP* plants (Fig 2A–2C). Measurements of growing rosette diameters from 8 days post-germination until bolting, confirmed that these genotypes are impaired in development and that the difference in growth starts to be visible as early as 8 days after germination (Fig 2C). Similar to *RNAi-AtTCTP* plants, the inflorescence stems of *AtCSN4-RNAi* plants were shorter and the plants exhibited a dwarf phenotype (Figs 2B and S5). Additionally, *RNAi-AtCSN4* plants had very short internodes between siliques resulting in a “bushy” plant phenotype (Figs 2B and S5).

Plants overexpressing *AtCSN4* (line *35S*::*AtCSN4*) had normal development and adult plants had similar size as WT Col-0 plants (Fig 2). Plants overexpressing *AtTCTP* (line *35S*::*AtTCTP*) exhibited accelerated growth and reached adult size earlier than the wild-type (Fig 2), in agreement with previously reported data [6]. The double overexpressor line (*35S*::*AtCSN4/35S*::*AtTCTP)* exhibited no additive effect and behaved as the single overexpressor line *35S*::*AtTCTP* (Fig 2).

Crosses between *RNAi-AtTCTP* and *AtCSN4* overexpressor lines on the one hand (line *RNAi-AtTCTP/35S*::*AtCSN4*) and between *RNAi-AtCSN4* and *AtTCTP* overexpressor lines on the other hand (*35S*::*AtTCTP/RNAi-AtCSN4*), yielded plants with delayed growth compared to the WT, thus a phenotype similar to the single *RNAi*-*AtTCTP* and *RNAi*-*AtCSN4* knockdown plants, respectively (Fig 2A–2C). We should note that *35S*::*AtTCTP/RNAi-AtCSN4* plants grew a little slower than *RNAi-AtCSN4*, and *RNAi*-*AtTCTP /35S*::*AtCSN4* plants grew a little faster than *RNAi-AtTCTP* plants. However, adult plants of the double transformant lines were indistinguishable from simple RNAi plants, demonstrating similar dwarf phenotype (Fig 2B). These data show that overexpression of *AtTCTP* or *AtCSN4* could not compensate the developmental anomalies induced by knockdown of *AtCSN4* or *AtTCTP*, respectively, and suggest that TCTP requires functional CSN4 to control growth. Despite several attempts, by plant genetic crossing or by genetic transformation, we were unable to generate the *RNAi-AtTCTP/RNAi-AtCSN4* double knockdown line. It is likely that double TCTP/CSN4 knockdown leads to plant lethality.

### AtTCTP and AtCSN4 regulate cell cycle progression and mitotic growth

**Fig 3:**
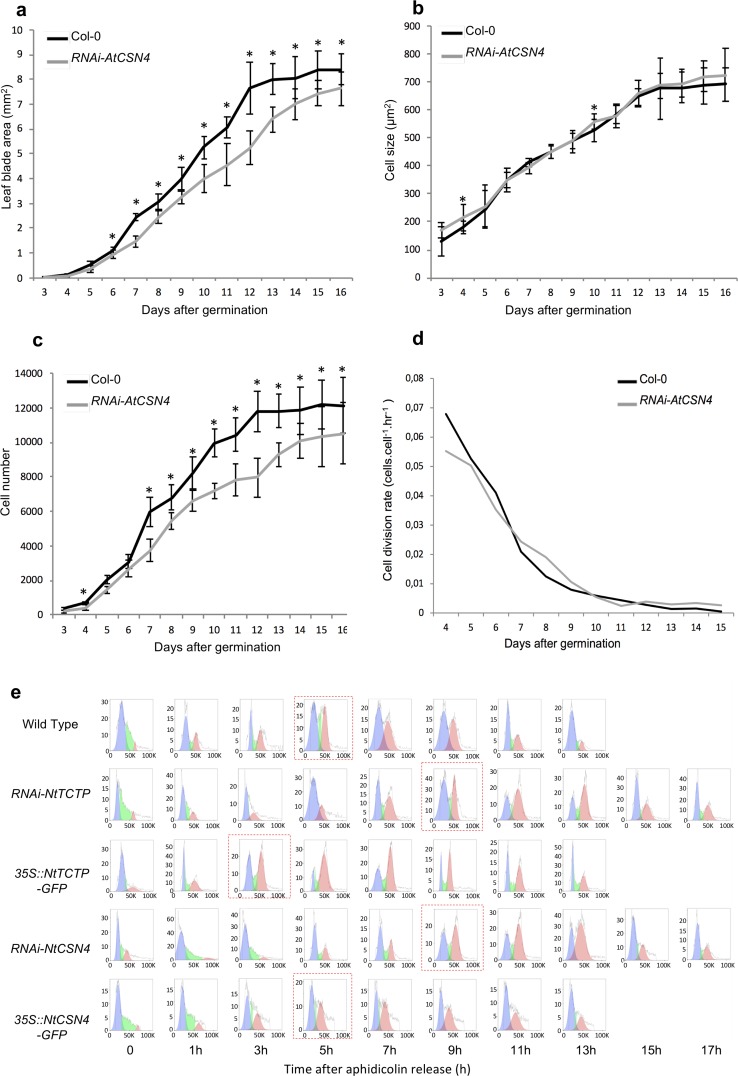
TCTP and CSN4 control cell proliferation and cell cycle progression. Leaf blade area **(a)** and cell number per leaf **(c)** are reduced in *RNAi-AtCSN4* plants compared to Col-0 WT due to a decrease in cell division rate **(d)**. Cell size in developing leaves **(b)** of *RNAi-AtCSN4* was the same as in Col-0 WT. n = 10; *: p-value <0,05. **(e)** Cell cycle progression at G1/S transition is slower in *RNAi-NtCSN4 and RNAi-NtTCTP* aphidicolin synchronized BY-2 cells compared to WT. The blue plots show cell population in G1 (2N), green plots are cells in S phase, and the red plots are cells in G2 (4N) phase.

To explore the cause of the growth defects observed in *RNAi-AtTCTP* and *RNAi-AtCSN4* plants, we analyzed cell division and cell expansion profiles. Previously, we demonstrated that downregulation of *AtTCTP* and the resulting decrease in plant organs size are correlated with reduced cell proliferation activity [6]. To explore if *AtCSN4* down-regulation is also associated with defects in cell proliferation, we performed kinematic analysis of leaf growth on plantlets grown *in vitro* [6,24]. Previously, kinematic of leaf growth analyses demonstrated that downregulation of *AtTCTP* results in smaller leaves compared to the WT, due to slower cell proliferation [6]. Similarly, in line *RNAi-AtCSN4* a significant reduction in leaf area was observed compared to the WT Col-0, starting from seven days after germination and the reduction was maintained during the whole observation period (Fig 3A). The reduction in leaf size correlated with about 20% decrease in cell number starting from day seven after germination and was maintained all along the observation period (Fig 3C), while no differences in cell size were observed (Fig 3B), thus again similar to *RNAi-AtTCTP* [6]. These data demonstrate that, similar to *AtTCTP*, the leaf growth defects associated with *AtCSN4* knockdown (*RNAi-AtCSN4*) correlated with the decrease in cell number, while cell size remained unchanged.

Using the slope of the log 2–transformed number of cell per leaf [25], we calculated the cell division rate in *RNAi-AtCSN4* plants. We observed that the cell division rate in *RNAi-AtCSN4* was slower than in WT Col-0 plants (Fig 3D) in the early stage of leaf development where the cell division activity is higher [26]. Previously, we reported a similar tendency for *RNAi-AtTCTP* lines [6]. Determination of the number of newly produced cells per hour during leaf development showed that the lower cell division rate in *RNAi-AtCSN4* line resulted in less newly produced cells at the beginning of leaf development (S6 Fig). Interestingly, the cell division rate was maintained at a higher level for longer time in the RNAi-AtCSN4, which indicates that a compensation mechanism likely exists. However, this was not enough to compensate the delay in leaf development, and therefore RNAi-AtCSN4 leaves stayed smaller than WT (Fig 3A). Later in leaf development, cell division in both plants was reduced to a very low level (Fig 3D).

We investigated root growth in both *RNAi-AtTCTP* and *RNAi-AtCSN4* lines. Similar to leaf growth, we also observed reduced root growth in developing seedlings of both lines (S7A Fig). Both *RNAi-AtTCTP* and *RNAi-AtCSN4* roots were shorter compared to WT starting as early as 3 days after germination (S7A Fig).

Similar to leaves and roots, petals of *RNAi-AtCSN4* were also smaller in size compared to WT Col-0 (S7B Fig). The petal size reduction was associated with reduced cell number, thus a phenotype similar to that observed in *RNAi-AtTCTP* (S7B Fig) [6]. These data corroborate the results obtained in leaves and demonstrate that, like for *AtTCTP*, downregulation of *AtCSN4* leads to reduced organ size as a result of altered cell proliferation. However, conversely to leaves, we observed that in petals of both *RNAi-AtCSN4* and *RNAi-AtTCTP* the defects in cell proliferation were associated with an increase in cell size (S7B Fig), suggesting a compensation mechanism in the petals. *AtTCTP* overexpression (line *35S*::*AtTCTP*) resulted in petals with increased size, but cell size was not affected, thus in agreement with Brioudes *et al*. [6]. *AtCSN4* overexpression (line *35S*::*AtCSN4*) did not affect petal development, (S7B Fig), thus corroborating the observed normal development of plants overexpressing AtCSN4 (Fig 2). Similar to observation during rosette development, overexpression of *AtTCTP* or *AtCSN4* in *RNAi-AtCSN4* and *RNAi-AtTCTP*, respectively, could not compensate for the petal developmental defects of the RNAi lines (S7B Fig). Line overexpressing both *AtTCTP* and *AtCSN4* showed similar phenotype to *35S*::*AtTCTP* (S7B Fig).

These data together show that the downregulation of *AtCSN4* leads to slower cell proliferation associated with reduced organs size, thus a phenotype similar to that observed in *AtTCTP* mutant plants.

To further identify the origin of this reduced cell proliferation activity, we investigated cell cycle progression in tobacco BY-2 cells down-regulating or overexpressing *NtCSN4* (line *RNAi-NtCSN4* and *35S*::*NtCSN4*, respectively) or *NtTCTP* (*RNAi-NtTCTP*, *35S*::*NtTCTP*, respectively). Both, CSN4 and TCTP proteins accumulated at low levels in *RNAi* BY-2 cells and over-accumulated in overexpressor BY-2 cells, as demonstrated by Western blot analysis (S8 Fig).

Wild-type BY-2 cells and BY-2 cells under- or over-accumulating TCTP (*RNAi-NtTCTP*, *35S*::*NtTCTP)* or CSN4 (*RNAi-NtCSN4*, *35S*::*NtCSN4)* were synchronized using aphidicolin. Cell cycle progression was followed every 2 hours after aphidicolin release (AAR) using flow cytometry (Fig 3E). Normal progression of cell cycle over time was observed in wild-type BY-2 cells with rapid reduction of G1 cells (2N) and a concomitant increase of G2 cells (4N) over the first 5 hours AAR, followed by a decrease of G2 cells with mitosis ending at about 13h AAR. In agreement with previously reported data [6], the G1/S transition in *RNAi-NtTCTP* BY-2 cells occurred with 4 hours delay compared to the wild-type and this delay was maintained all along the cell cycle (Fig 3E). Similarly, *RNAi-NtCSN4* BY-2 cells also showed a slower cell cycle progression with about the same 4 hours delay at the G1/S transition compared to wild-type BY-2 cells (Fig 3E). Like for *RNAi-NtTCTP*, the 4 hours delay of cell cycle progression in *RNAi-NtCSN4* was maintained until the end of the cell cycle, thus corroborating the kinematic of growth data obtained in *Arabidopsis* leaves (Fig 3).

These data together demonstrate that the downregulation of either *NtTCTP* or *NtCSN4* leads to comparable delays in cell cycle progression and that such delay occurs at G1 and/or early S phase.

Conversely to BY-2 cells knockdown for *NtTCTP*, BY-2 cells overexpressing *NtTCTP* (*35S*::*NtTCTP*) entered G1/S transition about 2 hours earlier than wild-type BY-2 (Fig 3E). However, these cells completed their first cell cycle the same time as WT BY-2 cells, at 13h AAR. This indicates that *35S*::*NtTCTP* BY-2 cells has longer S or M2 phase to compensate faster G1. In agreement with the absence of cell proliferation and developmental defects in *35S*::*AtCSN4 Arabidopsis* line (Figs 2 and S7B), no significant difference in cell cycle progression was observed in *35S*::*NtCSN4* BY-2 cells, compared to the wild-type (Fig 3E).

To further explore at which step of the cell cycle TCTP and CSN4 precisely act and to confirm BY-2 results directly *in planta*, we performed cumulative EdU labeling in the root tips of the different *Arabidopsis* lines to estimate S-phase length and total cell cycle length. Results show that, similar to observations in BY-2 cells, total cell cycle length in root tips was increased by 4 hours, both in *RNAi-AtTCTP* and *RNAi-AtCSN4* lines, while S-phase length remained unchanged (Table 1). Likewise, no substantial changes were observed in cell cycle length in *35S*::*AtTCTP* and *35S*::*AtCSN4* lines (Table 1), again consistent with the results obtained in BY-2 cells (Fig 3C). Interestingly, S-phases lengths again remained unchanged, suggesting that the observed cell cycle slow-down after the rapid progression through G1/S phase seen in *35S*::*NtTCTP* BY2 cells, did not affect S-phase but probably G2/M phase.

**Table 1:** TCTP and CSN4 does not control S-phase length during cell cycle progression.

		Cell Cycle Length (h)	Confidence Interval 95%	S-phase length (h)	Confidence Interval 95%
**1**	*Col-0*	15	[13,5–16]	1,5	[0,6–2,4]
	*RNAi-AtTCTP*	18**	[16,8–20]	1,5	[0,5–2,3]
	*RNAi-AtCSN4*	19***	[16,5–23,2]	1,9	[0,4–3,9]
	*35S*::*AtTCTP*	15	[13,5–15,8]	1,7	[0,9–2,5]
	*35S*::*AtCSN4*	15	[13,5–15,8]	1,5	[0,53–2,2]
**2**	*Col-0*	15	[13,6–15,9]	2,8	[0,9–3,1]
	*RNAi-AtTCTP*	19***	[16,4–20,3]	2,7	[0,8–3,2]
	*RNAi-AtCSN4*	19***	[16,5–23,1]	2,8	[1–3,7]
	*35S*::*AtTCTP*	15	[13,5–16]	2,8	[0,9–3,8]
	*35S*::*AtCSN4*	15	[12,8–16,1]	2,7	[0,8–3,7]

EdU incorporation in *Arabidopsis* root tips demonstrated in both *RNAi-AtTCTP* and *RNAi-AtCSN4* lines cell cycle duration is about 4h longer compared to Col-0 WT. Note that the length of S-phase was not affected in any of these lines. Data show results of two independent experiments. Asterisks indicate statistically significant differences (** p<0.01; *** p<0.001).

All these data together with the TCTP-CSN4 co-immunoprecipitation and the *in vivo* interactions studies suggest that TCTP and CSN4 interact physically to control G1/S transition during cell cycle progression.

### TCTP/CSN4 interaction impact CUL1^NEDD8^ /CUL1 ratio in plants and animals

**Fig 4:**
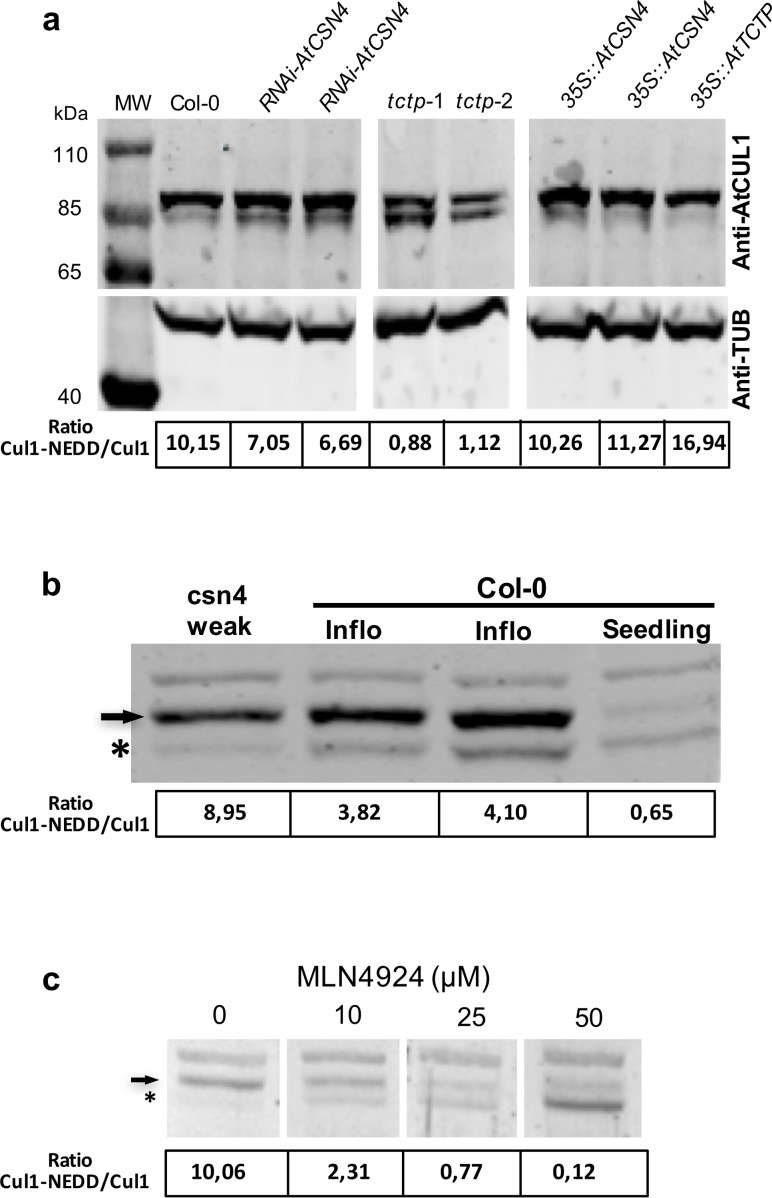
The CUL1^NEDD8^/CUL1 ratio is modified in Arabidopsis *tctp* mutants. (**a**) CUL1^NEDD8^/CUL1 ratio decreases in a similar manner in the two independent *tctp* knockout lines, *tctp-1* and *tctp-2*, while in plants overexpressing AtTCTP (*35S*::*AtTCTP*) increased CUL1^NEDD8^/CUL1 ratio was observed. **(b)** CUL1^NEDD8^/CUL1 ratio in inflorescence and seedlings of Col-0 WT plants and in inflorescence of weak mutant of *csn4*. **(c)** Treatment of WT Col-0 with MLN4924, a drug that inhibits neddylation, results in an increase of the free CUL1 form with concomitant decrease of the CUL1^NEDD8^ form, confirming that the observed bands correspond to CUL1 at different neddylation status. CUL1 protein was detected by Western blot using anti-CUL1 antibody. Quantifications of CUL1^NEDD8^/CUL1 ratio are shown under each lane. Anti-TUB is shown as control. Star: free CUL1; Arrow: CUL1^NEDD8^ form.

CSN4 is one of the eight subunits of the CSN that regulates CRL activity *via* the post-translational RUB/NEDD8 modification of its CUL subunit. To investigate the biological significance of AtTCTP/AtCSN4 interaction, we evaluated the neddylation status of the *Arabidopsis* CULLIN1 (CUL1), a major target of CSN. Using an antibody against CUL1, we were able to distinguish free CUL1 and the neddylated CUL1 (CUL1^NEDD8^) forms (Figs 4A–4C and S9A–S9C).

By growing plants on medium with increasing concentrations of MLN4924, a drug that inhibits CUL neddylation [27], we confirmed that the observed protein bands indeed correspond to CUL1 and CUL1^NEDD8^ (Figs 4C and S9C). In WT plants, we observed about 8–10 times more CUL1^NEDD8^ than free CUL1 while at 50μM MLN4924 we observed that almost all CUL1 were non-neddylated (Figs 4C and S9C). Moreover, we demonstrate that WT inflorescence contains more neddylated CUL1, while in WT seedlings non-neddylated CUL1 is more present (Figs 4B and S9B). Inflorescence of a weak mutant of *csn4* contains almost exclusively neddylated CUL1 (Fig 4B) in accordance with previous results [28].

Next, CUL neddylation status was analyzed in two independent *tctp* knockout lines (*tctp-*1 and *tctp-*2), in *35S*::*AtTCTP*, *RNAi*-*AtCSN4* and *35S*::*AtCSN4* (Figs 4A and S9A). For both *tctp*-1 and *tctp*-2, we observed a drastic decrease (Fig 4A) to complete absence (S9A Fig) of the CUL^NEDD8^, with a concomitant increase of free CUL1, leading to a drop in CUL1^NEDD8^/CUL1 ratio in knockout lines (Figs 4A and S9A). Overexpression of *AtTCTP* led to a slight increase in CUL1^NEDD8^ (Fig 4A) in agreement with the fact that although *35*::*AtTCTP* plants grow faster, fully adult plants showed a phenotype similar to the wild-type [6] (Fig 2). These data suggest a role for AtTCTP in the regulation of CUL1 neddylation status, which in turn influences the activity of CRL complexes. Only small changes of CUL1^NEDD8^/CUL1 ratio were observed in *RNAi-AtCSN4* plants (Fig 4A). This is likely due to the fact that flowers already accumulated high level of neddylated CUL1 (Fig 4B) and that in *RNAi-AtCSN4* lines we do not have full obliteration of AtCSN4 (S4A and S4B Fig).

In *35S*::*AtCSN4*, no decrease of CUL1^NEDD8^ was observed and the CUL1 neddylation status was similar to wild-type (Fig 4A). This is in agreement with previous studies showing that overexpression of only one subunit of the COP9 complex does not modify its deneddylation activity [29]. The data also corroborate the fact that CSN4 overexpression does not affect cell cycle progression as well as organ and plant development (Figs 2 and 3E and S7).

We demonstrated that CUL1 neddylation is affected in *tctp* mutants, suggesting that CRLs function in general might be affected. The best characterized SCF/CRL in *Arabidopsis* is SCF^TIR1^ implicated in auxin perception and signaling [30,31]. Therefore, we investigated if auxin responses are affected in *tctp* mutant. Auxin homeostasis reporter *DR5rev*::*GFP* and auxin efflux reporter *PIN1*::*PIN1-GFP* [32] were introgressed into *tctp* knockout plants. Similar to WT embryos, in *tctp* embryos *PIN1*::*PIN1-GFP* accumulated in apical cell lineage at globular and transition stage, then at heart stage the pattern switches to a PIN1 localization in the future cotyledons and across the provasculature (S10A Fig). In agreement with these data, in *tctp* mutant embryos the accumulation pattern of auxin homeostasis reporter *DR5rev*::*GFP* was similar to that of WT embryos. Moreover, the accumulation pattern of auxin homeostasis reporter *DR5rev*::*GFP* in response to exogenous treatment with synthetic auxin 2,4D was also similar in WT and *tctp* embryos (S10B Fig). These data show that the modification in CUL1 neddylation associated with *AtTCTP* mutation do not affect the auxin pathway nor the signaling via SCF^TIR1^, indicating that the role of TCTP-CSN4 interaction in regulating CUL neddylation and CRL activity is specific to cell cycle.

**Fig 5:**
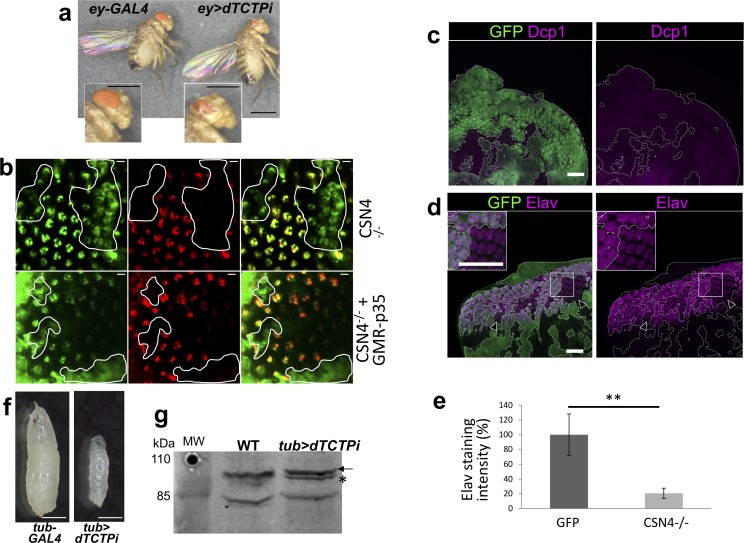
The role of TCTP and CSN4 in the control of CULLIN neddylation and cell proliferation is conserved in *Drosophila*. **(a)** Downregulation of *dTCTP* specifically in the eyes (*ey>dTCTPi*) of *Drosophila* leads to small eye phenotype. Close up images of wild type adult eye and of eye expressing *ey>dTCTPi* are shown (Bars = 500 μm). (**b**) *Drosophila* adult retina visualized by immersion microscopy with the Tomato/GFP-FLP/FRT method. CSN4 mutant clones are visualized by the expression of Rh1-GFP and by the absence of tomato (red) and are delineated by a white line. Photoreceptors are visualized by Rh1-GFP (Left panels), Rh1-tdTomato (Middle panels) or in the merge (Right panels). Photoreceptor absence is indicated by loss or diffuse GFP fluorescence in CSN4^k08018^ mutant clones (CSN4-/-, upper panels). CSN4^k08018^ mutant clones in which p35 is overexpressed (CSN4-/-, lower panels) still show loss or diffuse GFP staining in mutant clone. Bars = 5μm. (**c-e**) CSN4 mutant photoreceptors show a delay in the acquisition of neuronal identity but not in caspase staining. Third instar eye imaginal discs carrying *dCSN4*^*-/-*^ mutant clones visualized by the lack of GFP (green) Bars = 25 μm. (**c**) eye imaginal discs stained with anti-Dcp-1 (purple) show no increase of staining in *dCSN4*^*-/-*^ mutant clones. (**d**) Eye imaginal discs stained with anti-Elav show a delay of expression (arrowheads) at the morphogenetic furrow and a reduced level in *dCSN4*^*-/-*^ mutant clones. (**e**) Quantification of Elav staining in (**d**) shows significant reduction in *dCSN4*^*-/-*^ mutant clones compared to wild type (GFP) (n = 5; p < 0,01). (**f**) Downregulation of *dTCTP* in all tissues of *Drosophila* larvae (*tub*>*dTCTPi*) leads to developmental arrest with reduced size and subsequent larval lethality at the first instar larvae. 7 days after egg-laying, control larvae (*tub*-GAL4) are at third instar (left) while *tub*>*dTCTPi* larvae stay at first instar (right). Bars = 500 μm. **(g)** Impaired larval development is associated with a decrease of the CUL1^NEDD8^ abundance in the *tub*>*dTCTPi* compared to *tub*-GAL4. Star: free CUL1; Arrow: CUL1^NEDD8^ form.

Previously, we demonstrated that the role of TCTP in regulating cell proliferation was conserved between plants and animals. Furthermore, we demonstrated that AtCSN4 is able to interact with dTCTP (Figs 1A and S1C). Therefore, we investigated if the TCTP-CSN4 pathway has similar roles in *Drosophila* development as observed in plants. Using the UAS/GAL4 system [33], first, we generated flies in which the expression of *dTCTP* was silenced *via* the expression of a *dTCTP* RNAi under the control of the eye-specific promoter *eyless* (line *ey>dTCTPi*). *eyless* promoter drives gene expression in the eye imaginal disk anterior to the furrow at the time when eye cell progenitors are actively dividing [34]. Interference with *dTCTP* using the *eyless* promoter led to a significant size reduction of the eye, in agreement with Brioudes *et al* [6] (Fig 5A).

To study the role of CSN4 in the *Drosophila* eye, we used the P-element recessive lethal lines CSN4^k08018^ from the UCLA URCFG collection. Previous analysis of CSN4^k08018^reported a rough eye associated with loss of photoreceptor and patterning defects [35,36]. To further analyze the loss of dCSN4 function, we used the mosaic Tomato/GFP-FLP/FRT method in combination with the caspase inhibitor p35 [36,37]. In this system, the absence of red fluorescence (tdTomato) marks *dCSN4*^*-/-*^ mutant photoreceptors in a background where all photoreceptors express GFP in the adult *Drosophila* eye. This method allowed the observation of mosaic eyes, in which regions where the *dCSN4* gene have been inactivated can be detected by the absence of the tomato reporter (Fig 5B, middle panel). *dCSN4* inactivation led to a strong loss of photoreceptors as seen by the lack or diffuse GFP staining in *dCSN4*^*-/-*^ mutant clones of *Drosophila* adult retina (Fig 5B, left panel). Importantly, no effector caspase (dcp-1, death caspase-1) staining was detected in *dCSN4*^*-/-*^ mutant clones in third instar eye discs (Fig 5C). Moreover the loss of photoreceptor in *dCSN4*^*-/-*^ mutant clones was not rescued by the expression of the caspase inhibitor p35 (Fig 5B, lower panels), a protein that prevents apoptosis [38]. This indicates that the loss of photoreceptors in *dCSN4*^*-/-*^ mutant clones is not due to increased apoptosis but rather impaired proliferation as in *dTCTP* mutants.

Next, we examined the expression of the neuronal marker ELAV, which is progressively acquired in differentiating photoreceptor posterior to the morphogenetic furrow in third instar eye discs [34]. We observed a delay and a reduction of ELAV staining in *dCSN4*^*-/-*^ mutant clones compared to wild type (Fig 5D and 5E). The delay in the acquisition of ELAV marker in *dCSN4*^*-/-*^ mutant clones could be the consequence of a delay in the proliferation of dividing photoreceptor progenitors as previously described in *dachsund* mutant that allows a tight coordination of proliferation and differentiation [39].

To explore whether the effect of TCTP on CUL1 neddylation is also conserved between plants and animals, we investigated CUL1 neddylation in *Drosophila* knockdown for *dTCTP* under the control of *TUBULIN* constitutive promoter (line *tub>dTCTPi*). Interference with *dTCTP* under the *TUBULIN* constitutive promoter (*tub>dTCTPi)* led to severe larval developmental defects and to growth arrest after the first larvae instar (Fig 5F). We evaluated the CUL1 neddylation status in *tub>dTCTPi* larvae knockdown for *dTCTP* exhibiting severe developmental delay, as compared to the wild-type flies and we observed a drastic decrease of the CUL^NEDD8^ with a concomitant increase of free CUL1 (Fig 5G). These data strongly suggest that, like in *Arabidopsis*, knockdown of *dTCTP* or *dCSN4* led to impaired cell proliferation. Moreover, knockdown of *dTCTP* in *Drosophila* also affects CUL1 neddylation status in a similar manner than in *Arabidopsis*, suggesting a conserved role of TCTP in the control of CUL neddylation between plants and animals. Importantly, the fact that AtCSN4 interacts with dTCTP (Fig 1C) suggests that, similar to plant AtTCTP, *Drosophila* dTCTP controls CUL1 neddylation likely via its interaction with CSN4.

## Discussion

In plants and in animals, TCTP is known to be implicated in many cellular processes, but its mode of action is largely unknown. Previously, we demonstrated that in *Arabidopsis* and in *Drosophila*, TCTP has a conserved role in the control of organ growth by regulating cell proliferation. We showed that TCTP regulates cell cycle progression more specifically at the G1/S transition [6]. To gain insight into the pathway by which TCTP fulfills this function, we identified its interactors in *Arabidopsis*. Here, we establish a functional relationship between TCTP and CSN4, one of the eight subunits of the COP9 Signalosome, a complex conserved among eukaryotes [12].

Like *tctp* null mutants, *csn4* mutants are not viable [22] (and this study). We therefore analyzed partial loss-of-function lines using RNAi. Both *RNAi-AtTCTP* and *RNAi-AtCSN4 Arabidopsis* lines display dwarf phenotype and reduced organ size due to decreased cell proliferation, and more specifically to a delay in the G1/S transition as evidenced by tobacco BY-2 cell synchronization and EdU incorporation assays in *Arabidopsis* root meristematic cells.

The ability of AtTCTP and AtCSN4 to interact and the similarity of the phenotypes of RNAi lines suggest that they could function in the same pathway, which was corroborated by our genetic analyses. No additive phenotypic effect was observed in the double overexpressor and the down-regulation of AtCSN4 was epistatic on AtTCTP overexpression, and reciprocally, AtCSN4 overexpression did not fully rescue the developmental defects of *RNAi-AtTCTP* plants. However, we observed that although the *RNAi-AtTCTP* plants that overexpress *AtCSN4* exhibited delayed development compared to the wild-type, they grew faster than *RNAi-AtTCTP* plants. This could be due to the fact that *CSN4* is likely involved in other biological process required for plant development, separately from *AtTCTP* [40]. We were unable to generate *RNAi-AtTCTP/RNAi-AtCSN4* double knockdown line, likely because these plants are not viable. This could be due to the fact that both simple RNAi lines are only partially loss of function, and that simultaneous down-regulation of AtTCTP and AtCSN4 leads to defects comparable to what is observed in *tctp* or *csn4* knockout lines. However, we cannot rule out the possibility that AtTCTP and AtCSN4 could also be separately involved in several other biological processes required for plant development [3,4,6,40]. This is supported by the fact that *AtTCTP* and *AtCSN4* proteins are not always co-localized in the cell (Fig 1C). Thus, TCTP/CSN4 interaction is most likely required at a defined time points during cell proliferation, but both proteins have additional functions independently of their interaction.

Using synchronized BY-2 cells and EdU incorporation assays, we determined that both *RNAi-AtTCTP* and *RNAi-AtCSN4* present similar delays at the G1/S transition, reinforcing the idea that the two proteins act in the same pathway. On the other hand, adding more AtTCTP leads to accelerated cell cycle progression. The fact that no effect on cell cycle progression was observed in plants over-accumulating AtCSN4, suggests that AtTCTP is likely the limiting factor to control cell cycle progression in the AtTCTP-AtCSN4 pathway.

CSN4 is part of the COP9 complex known to control CRL *via* neddylation status of their CULLINS (CUL) subunits [17]. We therefore asked whether TCTP and CSN4 might control CUL neddylation status. Indeed, we observed that both *AtTCTP* and *dTCT*P misexpression strongly impacts CUL1^NEDD8^/CUL1 ratio supporting an elegant hypothesis relative to how TCTP controls G1/S transition. In fact, it is conceivable that *via* its interaction TCTP could sequester CSN4, preventing its association with the COP9 complex specifically at the G1/S transition. It is well established that the lack of one of the eight COP9 sub-units is sufficient to suppress the deneddylation activity [19,20,30,41]. Moreover, in *Drosophila* it was previously reported that during development, CSN4 functions to maintain self-renewal of stem cells, and the switch from self-renewal to differentiation requires the sequestration of CSN4 from the CSN by the protein Bam [42]. Another subunit, CSN5 was also demonstrated to be sequestered by the small protein Rig-G, in order to negatively regulate SCF-E3 ligase activity in mammalian cells [43]. We can thus imagine that a similar scenario exists between TCTP and CSN4 to drive cell cycle through the G1/S transition and maintain cell proliferation. This is the first time that a sequestration mechanism to regulate CSN activity is described in plants.

**Fig 6:**
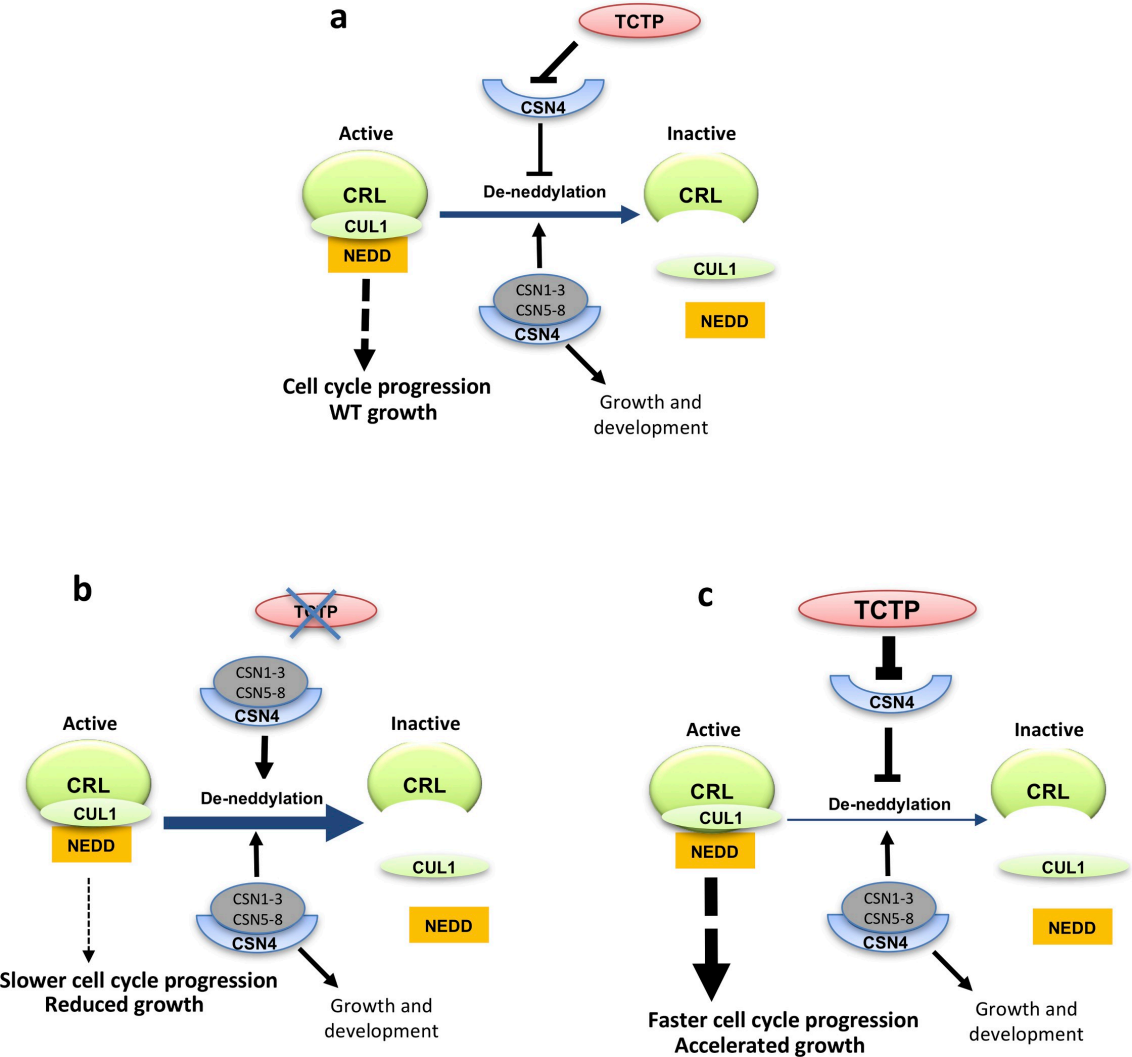
Model showing the interaction of TCTP and CSN4 and its impact on CUL neddylation and CRL complex and growth. (**a**) Wild type situation. (**b**) Mutation of TCTP leads to enhanced deneddylation of CUL and inactivation of CRL, resulting in slower cell cycle progression and reduced growth. (**c**) The inverse is observed when TCTP is overexpressed. Other pathways involving CUL neddylation *via* CSN are not shown. CSN1-3 and CSN5-8: subunits of the Cop9 complex forming the active COP9 complex when associated with CSN4.

This model is also consistent with the phenotypic defects triggered by TCTP deficiency (Fig 6). Franciosini *et al*. [44] suggested that during embryo maturation COP9 became deactivated, and subsequently reactivated at germination. It is possible that the role of TCTP during embryogenesis is to sequester CSN4 and prevent assembly and activity of COP9 complex during the G1/S transition. Moreover, it was demonstrated that CUL1 neddylation is increased during normal embryo development, thus the reduced neddylation in *tctp* embryos could explain their delayed development and death [6,44].

To our surprise, although *AtCSN4* down-regulation induced severe developmental defects, we were not able to detect strong modification in CUL1^NEDD8^/CUL1 ratio in *RNAi-AtCSN4* lines. This is probably due to the fact that CUL1^NEDD8^ level is already too high in the inflorescence to see a small increase due to reduced level of CSN4 accumulation and also to additional functions of CSN4 [16,18,40] independently of its interaction with TCTP. Also, because the CUL1^NEDD8^/CUL1 homeostasis is known to be highly dynamical process that is strictly regulated during plant development [44], it is possible that *AtCSN4* down-regulation in our RNAi lines is not strong enough to have measurable CUL1^NEDD8^ ratio changes at the studied developmental stages. However, we assumed that the developmental phenotypes observed in this line were due to compromised COP9 complex function. Indeed, previous work on dominant negative, weak alleles or RNAi lines of different CSN subunits demonstrated that the developmental defects, similar to those observed in our *RNAi-AtCSN4* line, were due to disturbance of CSN activity [28,31,41,44].

*TCTP* down-regulation leads to a decrease of the CUL1^NEDD8^/CUL1 ratio. Since CSN4 is involved in the deneddylation of CULs, its down-regulation would be expected to increase this ratio, as described in *csn4* mutant [22]. TCTP and CSN4 thus appear to act antagonistically on CULs neddylation, and one could therefore expect *RNAi-AtCSN4* and *RNAi-AtTCTP* lines to display opposite phenotypic defects instead of the similarities we report here. However, the effect of neddylation on CRLs activity is extremely complex: *csn* mutants that produce more neddylated CUL are expected to increase CRL activity resulting in positive effect on development. However, all *csn* mutants are delayed in their development. On the other hand, treatment of plants with MLN924, a drug that inhibits neddylation resulting in the accumulation of deneddylated CUL1, also leads to plant lethality [27], thus a similar phenotype as when TCTP is mutated. It is now well established that in addition to the CUL^NEDD8^/CUL ratio, the temporal kinetics of CUL and NEDD8 association/dissociation controls CRL activity [45–48]. Indeed, to be fully active, CRLs need to undergo a cycle of neddylation and de-neddylation [14,49] and inactivation of factors with opposite roles on this cycle can thus result in the same cellular defects. On the other hand, CRL acts both on positive and negative cell cycle regulators and the timing of the degradation of these regulators has to be tightly coordinated [16]. Thus, opposite perturbation of CRL activity by TCTP or CSN4 downregulation can lead to similar phenotypes.

Together, our results provide evidence for the role of TCTP and CSN4 in the control of cell cycle regulation *via* the modification of CUL1 neddylation status that affects CRL activity (summarized in Fig 6). However, the nature of the CRLs and their targets that account for this role on cell cycle regulation remains to be established.

In plants, the mechanisms controlling the G1/S transition are poorly known. However, it seems that there are close similarities compared to animals. Indeed, overexpression of CKI results in G1 arrest of the cell cycle in plants [50]. Furthermore, ICK2/KRP2, a CKI related protein, and other central cell cycle regulators such as E2Fc, a central transcription factor controlling G1/S transition in plants, must be degraded via the ubiquitin/26S proteasome pathway to allow the cells to go through the G1/S transition [51,52]. Although it was suggested that AtCSN4 is implicated in the G2/M transition [22], it has also been reported that G1/S phase specific core cell cycle genes were overexpressed in *csn4* mutant [22,53]. Furthermore, in human cells, it was reported that the downregulation of different CSN subunits can affect cell cycle in opposite ways, and the resulting perturbation of COP9 activity affect both G1/S and G2/M transition [54]. These published data suggest that CSN is likely equally important for G1/S and G2/M transitions in both plants and animals, and thus corroborate our findings here. They are also consistent with the fact that CUL deneddylation affects the activity of multiple CRL complexes.

Our data show that TCTP regulates G1/S transition, in agreement with Brioudes *et al* [6]. It is likely that TCTP/CSN4 interaction is specifically interfering with CSN function at G1/S. In this scenario, TCTP controls cell cycle through sequestration of CSN4, leading to CSN assembly impairment that in turn impacts neddylation status and stability of CRL complexes. CRLs have major role in several biological processes, among which hormone transduction and signaling are well studied [17]. The fact that no changes were observed in auxin flux and homeostasis in *tctp* knockout embryo during development is in favor of the conclusion that TCTP/CSN4 interaction likely controls CRLs specifically involved in cell cycle control but not in auxin signaling. It was reported that perturbation of CUL neddylation do not necessarily affect the activity of all CLRs. In mice, deletion of CSN8 did not affect SCF/CRL function in general, as a subset of CRLs maintained their capacity to degrade their substrate [55]. Similarly, in *Drosophila csn4* and *csn5* mutants, some but not all SCF/CRLs implicated in circadian rhythm maintenance are affected [56]. Therefore, it is likely that in *tctp* embryos, only a specific subset of CRLs implicated in cell cycle progression is affected, resulting in arrest of development, while auxin signaling remains normal. This is also supported by the fact that conversely to *tctp*, auxin signaling mutants are able to complete embryo development and produce mature seeds [57].

Previously, we demonstrated that TCTP function in the regulation of cell proliferation is conserved between plants and animals [6]. *Arabidopsis* AtTCTP and *Drosophila* dTCTP share only 38% amino acids identity, but many of the essential amino acids and domains known to be required for TCTP functions are conserved between plant and animal TCTPs [3,6]. Previously we showed that AtTCTP and dTCTP were able to homodimerize, but also to dimerize with each other *in vivo* [6], thus another argument that despite the overall relatively divergent protein sequences, their function is conserved. In support of a conservation and importance of TCTP/CSN4 interaction in animals, we show that, similar to *dTCTP* loss of function, *dCSN4* loss of function result in a loss of photoreceptors in adult *Drosophila* retina accompanied by a delayed acquisition of neuronal identity, which requires a tight coordination with cell proliferation in the developing eye disc. Furthermore, we show that, like for *AtTCTP*, down-regulation of *Drosophila dTCTP* also led to a decrease of CUL1^NEDD8^. Moreover, co-immunoprecipitation results show that dTCTP is able to interact with AtCSN4, which indicates that TCTP/CSN4 interaction is likely conserved between plants and animals.

In summary, our data provide evidences that TCTP functions as a key growth regulator by controlling cell proliferation together with CSN4. We propose that TCTP could sequester CSN4 to control CUL1 neddylation status and thus CRL activity (Fig 6), and this role is conserved between plants and animals. These data add a new piece to resolve the puzzle of developmental biology processes by connecting two evolutionary conserved pathways, TCTP and COP9, for cell proliferation. Our work will help future studies to better understand growth disorders and malignant transformation, associated with *TCTP* miss-expression.

## Materials and methods

### Constructs, plant biological material and growth conditions

T-DNA insertion knockout lines *tctp*-1 (SAIL_28_C03), *tctp*-2 (GABI_901E08) as well as the *RNAi-AtTCTP*, *35S*::*AtTCTP*, *35S*::*AtTCTP-GFP*, *35S*::*dTCTP-GFP* and the *AtTCTPg-GFP* lines harboring the *AtTCTP* genomic sequence including the promoter region, exons, introns, and the 3′ UTR region in which the GFP was inserted in frame with *AtTCTP* (At3g16640), have been previously described [6]. *csn4* knockout line (Salk_043720C) was provided by NASC. The weak allele mutant of AtCSN4 was kindly provided by C. Bellini (Umea University, Sweden).

*AtCSN4-RNAi* lines: The DNA fragment corresponding to the ORF of *AtCSN4* (At5g42970) without start codon ATG was cloned into the vector pB7GWIWG2D(II) [58] under the control of the CaMV 35S constitutive promoter. The resulting construct was then used to transform *A*. *thaliana* Col-0 plants. Lines are referred to as *RNAi-AtCSN4*.

*AtCSN4-GFP* overexpressing line: The DNA fragment corresponding to the ORF of *AtCSN4* was cloned into the pK7WGF2 [58] under the control of the CaMV 35S promoter. Lines are referred to as *35S*::*AtCSN4*.

The above lines were then crossed to generate the *35S*::*AtTCTP/RNAi-AtCSN4*, the *35S*::*AtCSN4/RNAi-AtTCTP* and the *35S*::*AtTCTP/35S*::*AtCSN4* lines.

*AtTCTPgGFP/35S*::*AtCSN4-Flag* line: The DNA fragment corresponding to the ORF of *AtCSN4* was cloned into pEarleyGate202 vector containing Flag motif [59]. The resulting construct was then used to transform *Arabidopsis* line *AtTCTPg-GFP* that harbors *pTCTP*::*TCTPg-GFP* construct [6].

*Arabidopsis thaliana* Columbia-0 (Col-0) was used as the wild-type (WT) for all experiments.

Embryo rescue of homozygous *tctp-1* and *tctp-2* embryos was performed as described previously [6]. During all steps, embryos from wild type siliques were used as control.

*PIN1*::*PIN1-GFP/TCTP*^*+/-*^ and *DR5rev*::*GFP/TCTP*^*+/-*^ were generated by crossing *tctp-*2^*+*/-^ with *PIN1*::*PIN1-GFP* or *DR5rev*::*GFP*, respectively. Embryo from white seeds and green seeds were then isolated [6] and observed under LSM 710 confocal microscope (Zeiss). *In vitro* culture of *Arabidopsis* embryo and hormone treatments with 2,4D were performed as previously described [60].

All seedlings were grown in culture chambers under shorts-day condition (8h/16h day/night at 22°C/19°C) for 4 weeks, then transferred to long-day condition (22°C, 16h/8h light/dark) to promote flowering, with light intensity of 70 μEm^-2^ sec^-1^.

### Fly analyses

All flies were maintained on standard corn/yeast medium at 25°C.

*eyless>dTCTPi* line: Expression of *dTCTPi* was carried out using the GAL4/UAS expression system [33]. We crossed *eyless*-GAL4 line (Bloomington Drosophila Stock Center) with UAS::dTCTPi [10] and analyzed the F1 adult progeny.

*tub>dTCTPi* line: Expression of *dTCTPi* was carried out using the GAL4/UAS expression system. We crossed *tub*-GAL4 line (Bloomington Drosophila Stock Center) with UAS::dTCTPi [10] and analyzed the F1 larvae.

We generated *dCSN4* mosaic clones in the eye using Tomato/GFP-FLP/FRT method [36,37]. The following fly strains were used: P{neoFRT}42D P{lacW}CSN4^k08018^/CyO (Kyoto Center), ey-FLP; FRT42D, Rh1-tdTomato[ninaC]/CyO; GMR-p35,Rh1-GFP/TM6B and ey-FLP; FRT42D, Rh1-tdTomato 94.1/CyO; UAS-GFP. Living adult flies were anesthetized using CO_2_ and embedded in a dish containing 1% agarose covered with cold water, as described [37] and imaged using a Leica SP5 upright confocal microscope using a water immersion objective. Photoreceptors were marked by Rh1-GFP or Rh1-tdTomato.

Mosaic clones were also generated using ey-FLP; FRT42D Ubi-GFP and eye discs analyzed at third instar larvae as previously described [61]. Dissected eye discs were stained with a rat anti-ELAV (Developmental Hybridoma Bank, 1/10) or anti-Dcp-1 (Cell Signaling, 1/300) and a rabbit anti-GFP (Invitrogen, 1/400) and imaged using a LSM800 Zeiss confocal microscope.

### BY-2 cell lines

*RNAi-NtTCTP* and *35S*::*NtTCTP-GFP* BY-2 cell lines have been described previously [6].

*RNAi-NtCSN4* and *35S*::*NtCSN4-GFP* BY-2 cell lines: DNA corresponding to *NtCSN4* ORF was amplified using primers BY2-CSN4-F (CACCATGGAGAGTGCGTTCGCTAGTG) and BY2-CSN4-R (CTAGACAGGAATAGGGAGCCCCTTCT) and cloned into the vector pK7GWIWG2 or pK7WGF2, respectively [58]. The resulting constructs were used to transform tobacco BY-2 (*N*. *tabacum* L. cv. Bright Yellow-2) cell suspension as previously described [62]. BY-2 cells were grown in the dark at 25°C constant temperature and with agitation at 150 rpm.

### Cell cycle synchronization of BY-2 cells and DNA content analyses

BY-2 cells were synchronized using aphidicolin (Sigma) as previously described [6]. Samples were collected every two hours and flow cytometry analyses were performed essentially as previously described [6]. Fluorescence intensity of stained nuclei was measured with MACSQuant VYB flow cytometer (BD Bioscience), using 405nm excitation blue laser. DNA content analysis was performed using FlowJo,LLC software version 10.

### EdU incorporation assay

Seeds of the relevant lines were germinated on half strength MS. Five days after germination, plantlets were transferred to EdU (10 μM, Sigma-Aldrich) supplemented medium and harvested after 3h, 6h, 9h and 12h of incubation. Experiments were performed as described [63]. The percentage of EdU positive nuclei increases linearly with time, and follows an equation that can be written as *P* = *at* + *b* where *P* is the percentage of EdU positive nuclei and *t* is time. Total cell cycle length is estimated as 100/*a*, and S phase length is *b/a*. The parameters of the equation and their confidence intervals were estimated with the R statistics software using the least-square method.

### Plant and organ growth analyses

Kinematic of leaf growth was performed as previously described [24] on the two first initiated-leaves of Col-0 WT and *RNAi-AtCSN4* plants grown *in vitro*. Leaf size as well as number and size of abaxial epidermal cells were determined starting of day 3 and until day 16 after germination. The average cell division rates were determined by calculating the slope of the Neperian Logarithmic-transformed number of cells per leaf, which was done using five-point differentiation formulas [25]. The number of newly produced cells were calculated by 72h time period.

Rosette diameter was determined starting of 8 days after germination, until bolting. Rosette area was measured every 3 days with a caliper. Each measure was performed using 18 plants for each genotype. Experiments were performed on two independent transformation events and in three biological replicates.

To investigate root growth, seeds were germinated on half strength MS and 5 day-old plantlets were transferred to a new plate and grown vertically for 6 days. Root length of each plant was measured using the Fiji software at day 0, 3 and 6 after transfer.

Petal area, petal cell number and size measurements were performed as previously described [64]. Briefly, petals were cleared overnight in a solution containing 86% ethanol and 14% acetic acid followed by two incubations of 4h each in ethanol 86%. Petals were dissected and photographed using Leica MZ12 stereomicroscope. Cells from cleared petals were observed with a Nikon Optiphot 2 microscope with Nomarski optics. Petal area and cell density i.e. number of cells per surface unit, were determined from digital images using ImageJ software (U. S. National Institutes of Health).

### Proteins interactions *in planta*

Co-localization experiments: cDNA fragments corresponding to the coding sequences of *AtTCTP* and *AtCSN4* were PCR amplified and then fused to RFP and GFP, respectively. Resulting constructs were used to infiltrate leaf epidermal cells of *Nicotiana benthamiana* plants as previously described [65].

Bimolecular Fluorescence Complementation (BiFC) experiments: cDNA fragments corresponding to the coding sequences of *AtTCTP* and *AtCSN4* were PCR amplified and then cloned into the pBiFP1, pBiFP2, pBiFP3 and pBiFP4 vectors [66] using the Gateway technology. Resulting constructs were used to infiltrate leaf epidermal cells of *Nicotiana benthamiana* plants as previously described [65]. BiFC was observed four days post-infiltration using a LSM 710 Confocal microscope (Zeiss).

### Immunoprecipitation

Co-immunoprecipitation experiments using AtTCTP-GFP or AtCSN4-GFP as bait were performed using the μMACS GFP Isolation Kit (Miltenyi Biotec). Three biological replicates were performed for each sample. Wild-type Col-0 and 35S::GFP plants were used as controls. Tissues of 10 days-old seedlings, mature seeds harvested from green siliques or inflorescences were ground to a fine powder in liquid nitrogen using mortar and pestle. The tissue powder (200 mg) was resuspended with 1ml pre-cooled (4°C) Miltenyi lysis buffer complemented with one tablet of cOmplete Mini EDTA-free Protease Inhibitor Cocktail (Roche) for 5 ml of lysis buffer. Cellular extracts were incubated on ice 10 min and then centrifuged 10 min at 21 000g (4°C). The supernatants were incubated with 50μl anti-GFP antibody coupled to magnetic μMACS microbeads for specific isolation of GFP-tagged protein during 1 hour at 4°C on orbital shaker. Microbeads were bound to magnetic columns and washed as described by the manufacturer, before elution of GFP-tagged proteins and bound proteins. Eluted proteins were analyzed by Western blot. For the immunoprecipitation followed by mass spectrometry (IP/MS) we visualized proteins by silver staining of the SDS-PAGE gel (ProteoSilver Plus Silver Stain Kit, SIGMA).

### Protein extraction and western blot analysis

Tissue (plants or cell culture) were grinded and total proteins were extracted using « Plant Total Protein Extraction Kit » (Sigma).

Protein from *Drosophila* larvae were extracted using approximately 10 larvae in 100 μl of protein extraction buffer (20 mM HEPES pH:7,5, 100 mM KCl, 5% Glycerol, 10 mM EDTA, 0,1% Tween, 1 μM DTT, 1 μM PMSF, 5 μl/ml Protease Inhibitor Cocktail (Sigma), 5 μl/ml Phosphatase Inhibitor (Sigma)). After centrifugation for 10 min at 160000 g, proteins contained in the supernatant were dosed using Bradford method[67].

Proteins were analyzed by Western blot using antibodies directed against AtTCTP (1/500 dilution) [6], AtCUL1 (1/2000 dilution; Enzo LifeScience), dCUL1 (1/500 dilution; Thermo Scientific), AtCSN4 (1/2000 dilution; Enzo LifeScience), Flag (1/1000 dilution; Sigma-Aldrich); α-Tubulin (1/1000 dilution; Sigma) or GFP (1/2000 dilution; Roche). IRDye 800CW and IRDye 680RD (1/10 000 dilution; LI-COR) were used as secondary antibodies and the signal was revealed using Odyssey Clx imaging system and signal intensity was quantified using the Image Studio Lite software (LI-COR). HRP conjugated anti-mouse or anti-rabbit IgG were used as secondary antibodies (1/5000 dilution) and the signal was revealed using Clarity Western ECL substrate and the ChemiDocTouch imaging system (Biorad). Intensity of the bands was quantified using ImageJ software (U. S. National Institutes of Health).

## Supporting information

S1 Fig AtTCTP and AtCSN4 interact *in vitro* and *in vivo*. **(a-c)** TCTP interacting proteins were co-immunoprecipitated from protein extracts prepared from seedlings **(a)** or from mature green seeds **(b)** of *AtTCTPg-GFP/35S*::*AtCSN4-Flag* plants, and from inflorescences **(c)** of *35S*::*GFP*, *AtTCTPg-GFP* (two independent lines 1 & 2), *35S*::*AtTCTP-GFP* and *35S*::*dTCTP-GFP*/*tctp* (two independent lines 1 & 2) plants, using anti-GFP coupled magnetic beads. Co-immunoprecipitated proteins were detected by Western blotting using anti-Flag (**a,** lower panel; **b**, left panel), anti-GFP (**a,** upper panel; c, lower panel), anti-TCTP (**c,** middle panel) or anti-CSN4 (**b,** right panel; **c**, upper panel) antibodies. Red asterisks: CSN4 protein; white arrows: CSN4-Flag protein; black arrows: TCTP-GFP protein; black asterisks: free GFP.
**(d)** CSN4 interacting proteins were co-immunoprecipitated from protein extracts prepared from inflorescences of Col-0, *35S*::*GFP* and *35S*::*AtCSN4-GFP* plants using anti-GFP coupled magnetic beads. Co-immunoprecipitated proteins were detected by Western blotting using anti-CSN4 (upper panel), anti-TCTP (middle panel) or anti-GFP (lower panel) antibodies. Red asterisks: CSN4 protein; white arrows: CSN4-GFP protein; blue arrows: TCTP protein; black arrows: TCTP-GFP protein; black asterisks: free GFP.
(TIF)

S2 Fig AtTCTP and AtCSN4 homodimerise *in vivo*. **(a)** Bimolecular fluorescence complementation assays show that AtTCTP or AtCSN4 fused with N- and C-terminal YFP moieties are able to form homodimers. No signal was observed in the control assays in which AtTCTP or AtCSN4 fused with N- or C-terminal YFP moieties was co-infiltrated with an empty plasmid (**b, c**; respectively).
(TIF)

S3 Fig *csn4* exhibits constitutive photomorphogenesis and severe delay in seedling development. Wild type Col-0 and *csn4* seedlings grown in light **(a)** or dark **(b)** show severe developmental delay. Plants at 10 days after germination are shown. *csn4* seedlings grown in dark show no hypocotyl elongation **(b)**, confirming the constitutive photomorphogenesis phenotype. Bars = 500μm.
(TIF)

S4 Fig Quantification of AtTCTP and AtCSN4 accumulation. AtCSN4 **(a,b)** and of AtTCTP **(c,d)** protein accumulation was assessed by Western blot in the different plant lines downregulated and/or overexpressor of AtCSN4 or AtTCTP.
Relative AtCSN4 or AtTCTP accumulation in the different plant lines was determined compared to accumulation in the WT Col-0 (= 1). Values are shown under each lane.
Black arrow indicates AtCSN4-GFP. Red arrow indicates endogenous AtCSN4. Blue arrow: AtTCTP. *: α-Tubulin (TUB) was used as loading control.
(TIF)

S5 Fig *RNAi-AtCSN4* and *RNAi-AtTCTP* inflorescence phenotype. *RNAi-AtCSN4* and *RNAi-AtTCTP* plants exhibit similar dwarf phenotype of flower stem with short internodes. Bars = 1cm.
(TIF)

S6 Fig Reduced cell division during leaf development in *RNAi-AtCSN4* line. The number of newly produced cells per hour was reduced in *RNAi-AtCSN4* plants compared to Col-0 WT. The number of newly produced cells was determined by 72h period. The error bars represent standard errors. n = 10; *: p-value <0,05.
(TIF)

S7 Fig Root growth, and petal size and cell size measurements. **(a)** *RNAi-AtTCTP* and *RNAi-AtCSN4* plants exhibit reduced root growth compared to the wild-type (Col-0). Root length was measured at day 5, 8 and 11 days after germination. Values are average +/- standard error (n = 30 for *RNAi-AtTCTP* and n = 20 for *RNAi-AtCSN4*). Asterisks indicate statistically relevant differences (T-test; p-value <0.01).
**(b)** Compared to the WT, mature petals of lines *tctp*, *RNAi-AtTCTP*, *RNAi-AtCSN4*, *RNAi-AtTCTP/35S*::*AtCSN4* and *35S*::*TCTP/RNAi-AtCSN4* are reduced in size with increased cell size, suggesting lower cell division rate.
Conversely, mature petals of lines overexpressing AtTCTP (lines *35S*::*AtTCTP)* and the double overexpressor *35S*::*AtTCTP/35S*::*AtCSN4* are larger in size while cell size was unaffected or smaller, respectively, compared to Col-0. This suggest increased cell division rate in these lines. The stars indicate significant differences relative to the WT Col-0 (T-test; p-value < 0,001).
(TIF)

S8 Fig NtTCTP and NtCSN4 accumulation in BY-2 cell lines. Western blot assay to evaluate the accumulation of NtTCTP **(a)** and NtCSN4 (**b)** in WT BY-2 tobbacco cells, and in BY-2 cells knockdown and overexpressor for these genes.
The relative accumulation of NtTCTP and NtCSN4 based on Western blot data is shown under each lane. Black arrows indicate GFP fused proteins (NtTCTP-GFP or NtCSN4-GFP). Red arrows indicate endogenous NtTCTP and NtCSN4 proteins.
(TIF)

S9 Fig CUL1 neddylation is modified in *tctp* mutant lines. **(a)** CUL1 neddylation is decreased in *tctp* mutants. Three independent samples (1–3) were analyzed using two independent *tctp* knockouts (*tctp-*1 and *tctp-*2). **(b)** CUL1^NEDD8^/CUL1 ratio in inflorescence and seedlings of Col-0 plants. **(c)** Treatment with MLN4924, a drug that inhibits neddylation, results in an increase of the free CUL1 form with concomitant decrease of the CUL1^NEDD8^ form, confirming that the observed two bands correspond to neddylated and non neddylated CUL1. CUL1 protein was detected by Western blot using anti-CUL1 antibody. Quantification of CUL1^NEDD8^/CUL1 ratio is shown under each lane. Star: CUL1. Arrow: CUL1^NEDD8^.
(TIF)

S10 Fig Auxin transport and accumulation is not modified in *Arabidopsis tctp* mutants. **(a)** PIN1::PIN1-GFP localization in *tctp* knockout embryos is similar to that in WT embryos, indicating that auxin efflux is not disturbed by *tctp* loss-of-function. Embryos at globular, transition and heart stages are shown. Bars: 2 0μm.
**(b)** The accumulation of GFP, expressed under the control of synthetic auxin response *DR5rev* promoter, is not disturbed in *tctp* mutant embryos compared to WT embryos, indicating that auxin transduction pathway is not disturbed by *tctp* loss-of-function. Exogenous treatment with synthetic auxin, 2,4-D leads to similar expansion of DR5rev-GFP expression in *tctp* mutant and WT embryos. Bars = 20 μm.
(TIF)

S1 File File containing numerical data underlaying the graphs in Figs 2, 3, 5 and S6 and S7. (XLSX)
