# Supplementary figures and images for: TCTP and CSN4 control cell cycle progression and development by regulating CULLIN1 neddylation in plants and animals
Source: PLoS Genet. 2019 Jan 29;15(1):e1007899. doi: 10.1371/journal.pgen.1007899 (PMC6368322; doi:10.1371/journal.pgen.1007899)

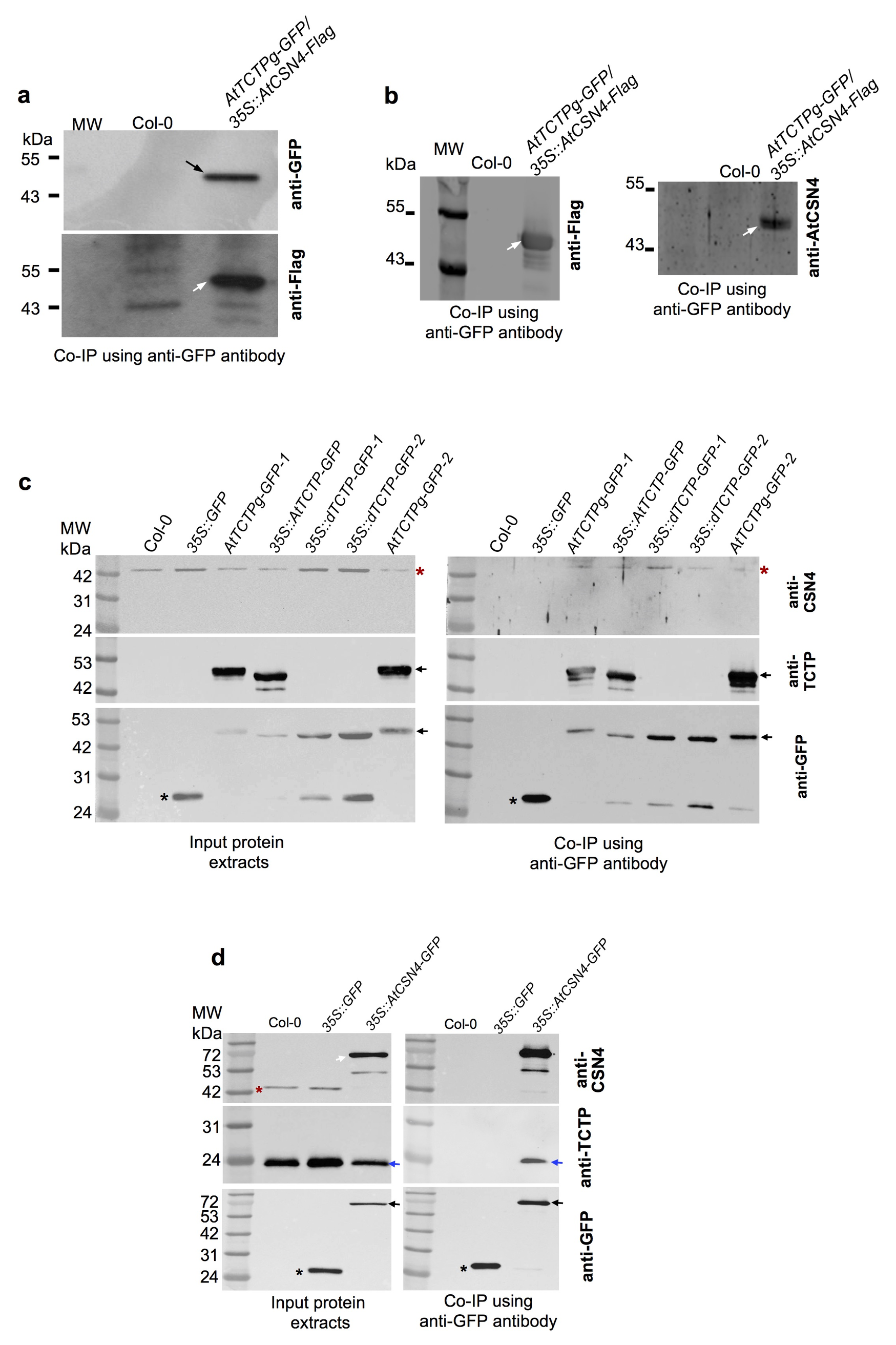

Supplement: S1 Fig — (a-c) TCTP interacting proteins were co-immunoprecipitated from protein extracts prepared from seedlings (a) or from mature green seeds (b) of AtTCTPg-GFP/35S::AtCSN4-Flag plants, and from inflorescences (c) of 35S::GFP, AtTCTPg-GFP (two independent lines 1 & 2), 35S::AtTCTP-GFP and 35S::dTCTP-GFP/tctp (two independent lines 1 & 2) plants, using anti-GFP coupled magnetic beads. Co-immunoprecipitated proteins were detected by Western blotting using anti-Flag (a, lower panel; b, left panel), anti-GFP (a, upper panel; c, lower panel), anti-TCTP (c, middle panel) or anti-CSN4 (b, right panel; c, upper panel) antibodies. Red asterisks: CSN4 protein; white arrows: CSN4-Flag protein; black arrows: TCTP-GFP protein; black asterisks: free GFP. (d) CSN4 interacting proteins were co-immunoprecipitated from protein extracts prepared from inflorescences of Col-0, 35S::GFP and 35S::AtCSN4-GFP plants using anti-GFP coupled magnetic beads. Co-immunoprecipitated proteins were detected by Western blotting using anti-CSN4 (upper panel), anti-TCTP (middle panel) or anti-GFP (lower panel) antibodies. Red asterisks: CSN4 protein; white arrows: CSN4-GFP protein; blue arrows: TCTP protein; black arrows: TCTP-GFP protein; black asterisks: free GFP. (TIF) [file pgen.1007899.s001.tif]

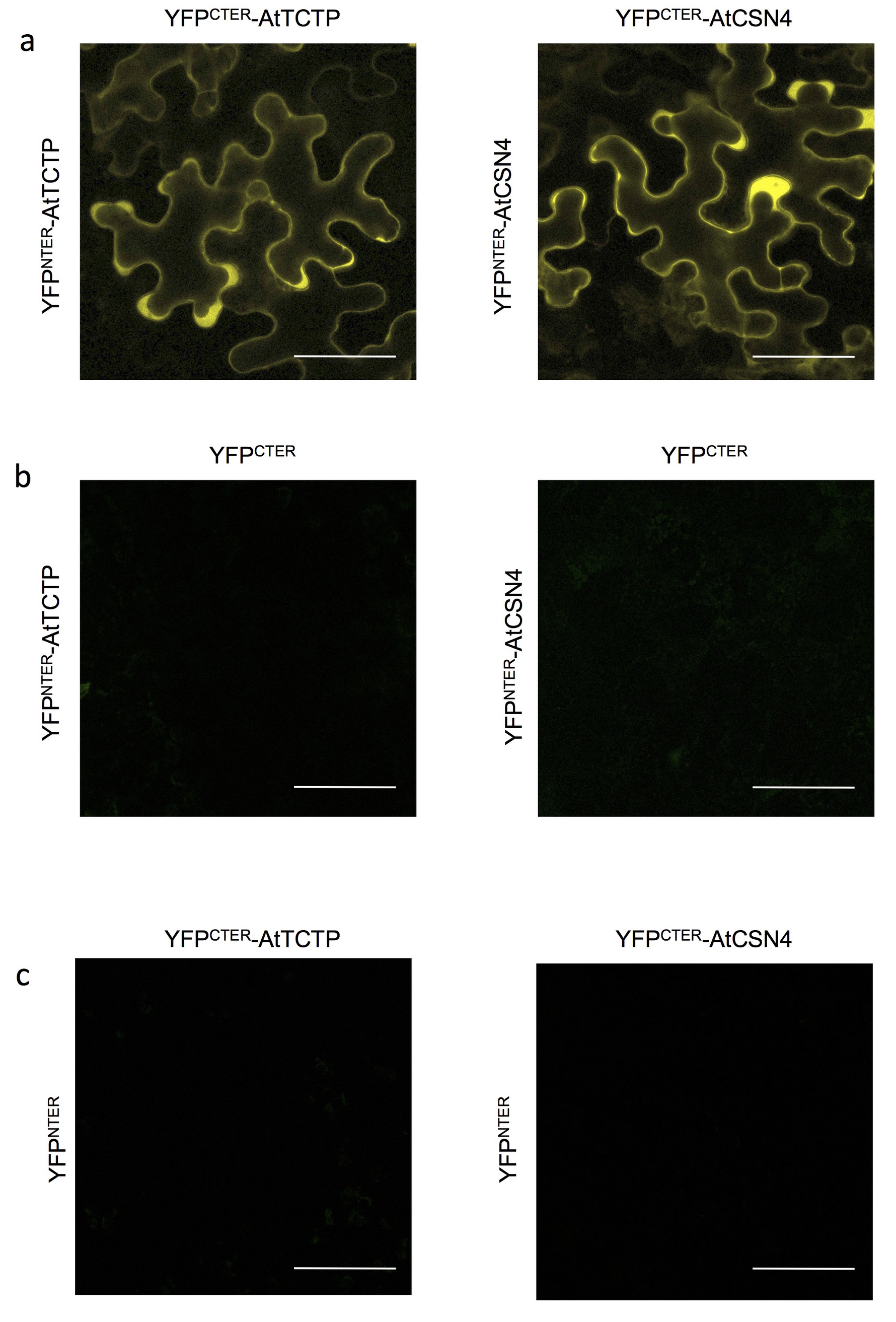

Supplement: S2 Fig — (a) Bimolecular fluorescence complementation assays show that AtTCTP or AtCSN4 fused with N- and C-terminal YFP moieties are able to form homodimers. No signal was observed in the control assays in which AtTCTP or AtCSN4 fused with N- or C-terminal YFP moieties was co-infiltrated with an empty plasmid (b, c; respectively). (TIF) [file pgen.1007899.s002.tif]

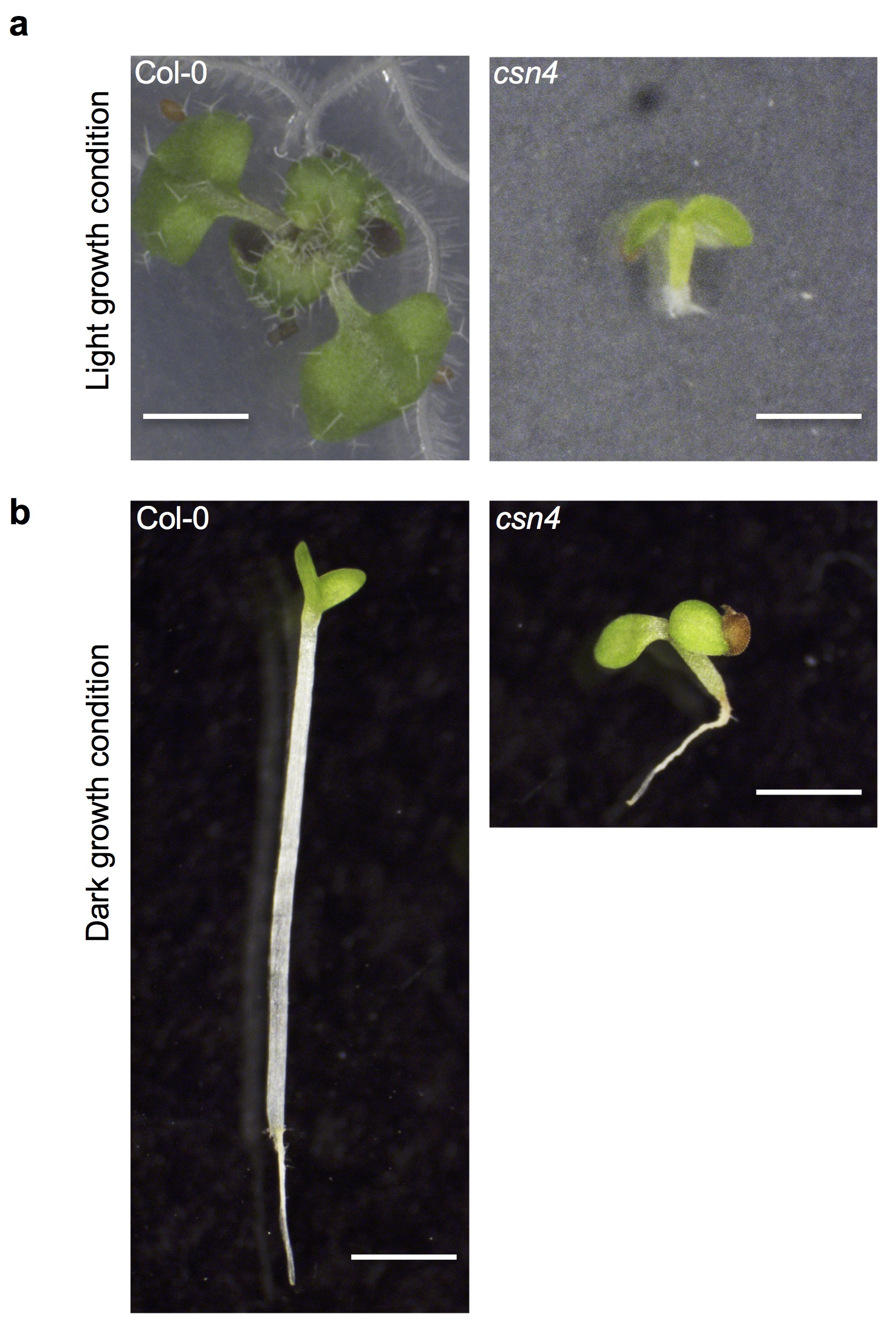

Supplement: S3 Fig — Wild type Col-0 and csn4 seedlings grown in light (a) or dark (b) show severe developmental delay. Plants at 10 days after germination are shown. csn4 seedlings grown in dark show no hypocotyl elongation (b), confirming the constitutive photomorphogenesis phenotype. Bars = 500μm. (TIF) [file pgen.1007899.s003.tif]

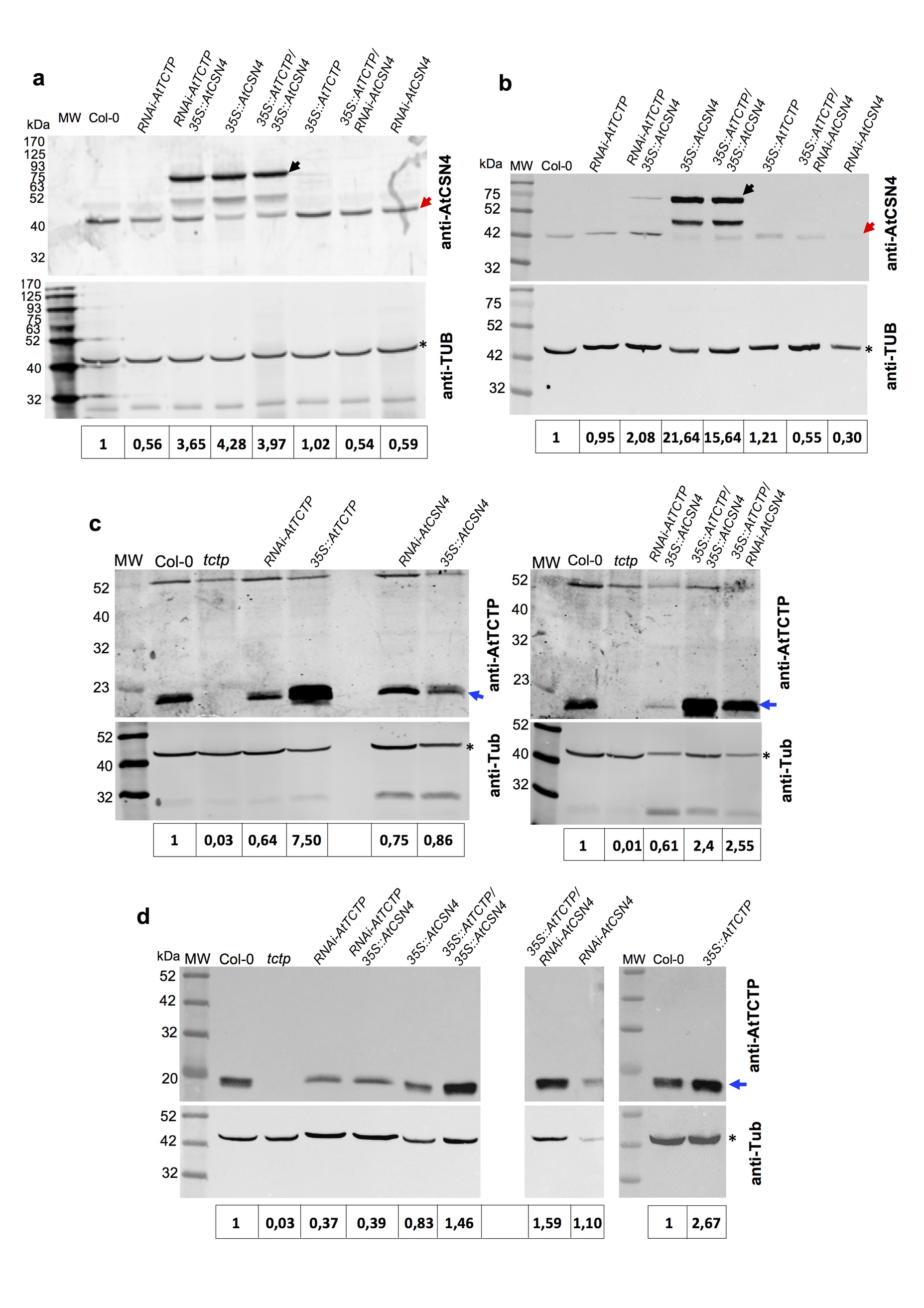

Supplement: S4 Fig — AtCSN4 (a,b) and of AtTCTP (c,d) protein accumulation was assessed by Western blot in the different plant lines downregulated and/or overexpressor of AtCSN4 or AtTCTP. Relative AtCSN4 or AtTCTP accumulation in the different plant lines was determined compared to accumulation in the WT Col-0 (= 1). Values are shown under each lane. Black arrow indicates AtCSN4-GFP. Red arrow indicates endogenous AtCSN4. Blue arrow: AtTCTP. *: α-Tubulin (TUB) was used as loading control. (TIF) [file pgen.1007899.s004.tif]

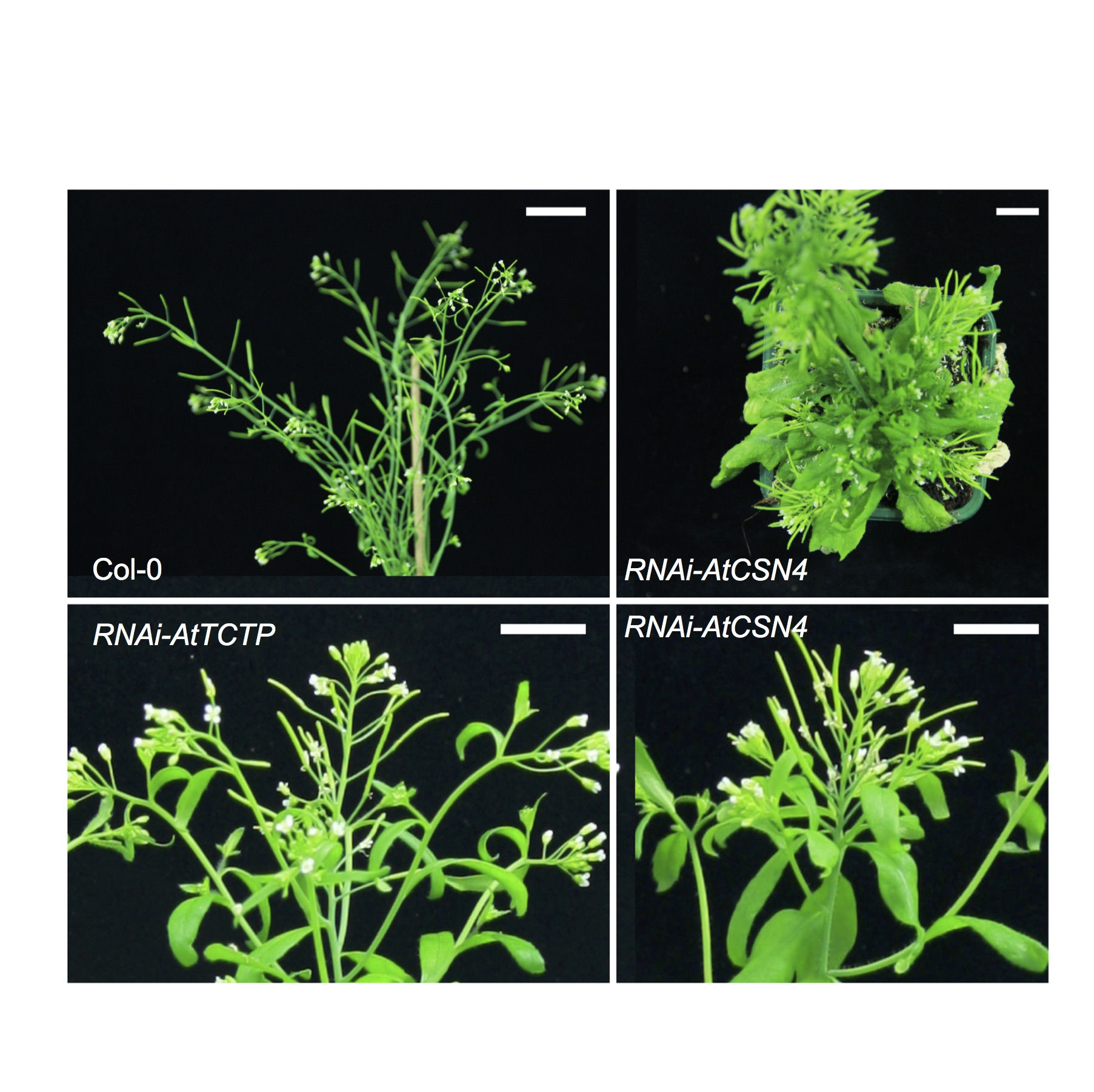

Supplement: S5 Fig — RNAi-AtCSN4 and RNAi-AtTCTP plants exhibit similar dwarf phenotype of flower stem with short internodes. Bars = 1cm. (TIF) [file pgen.1007899.s005.tif]

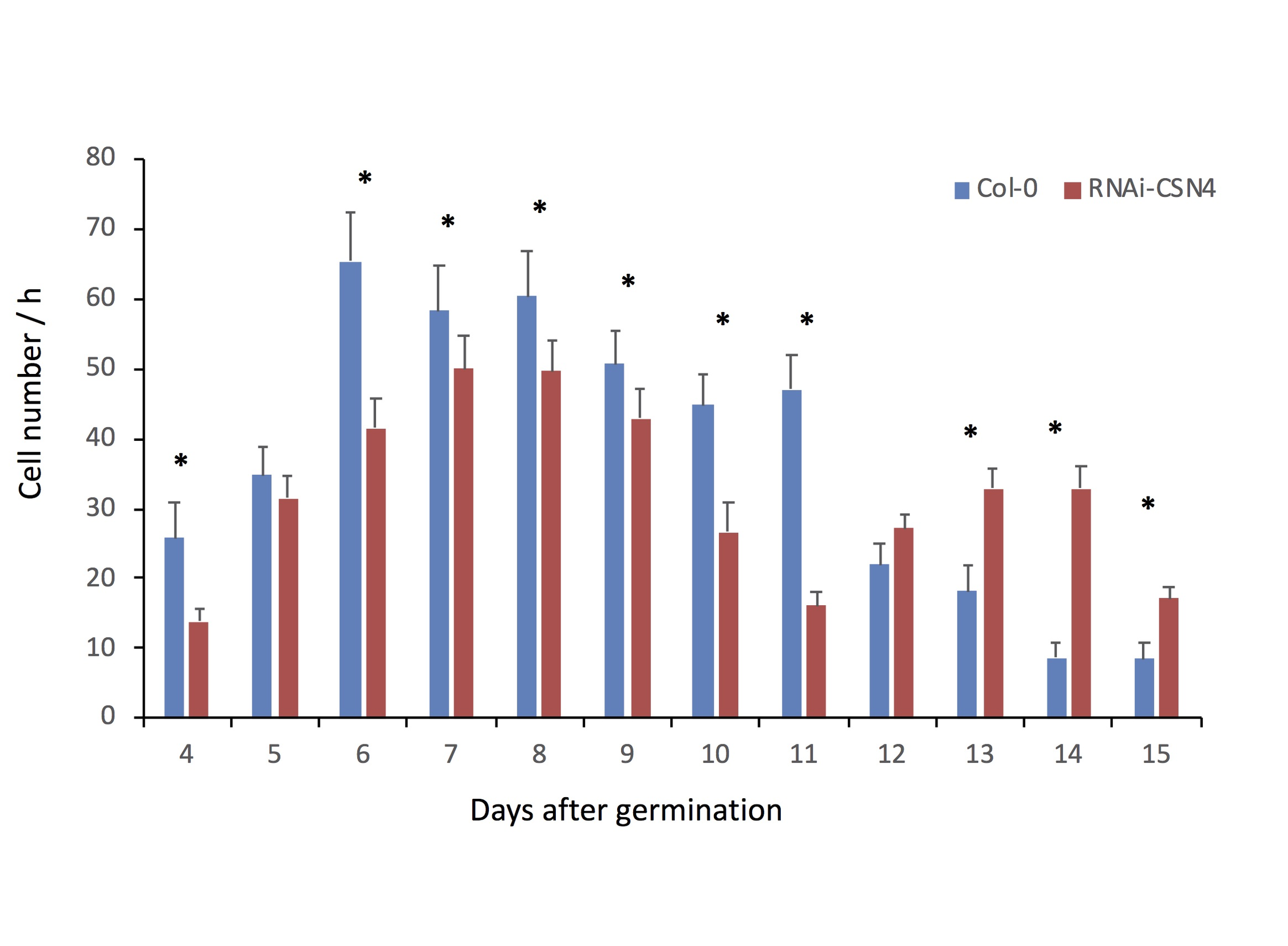

Supplement: S6 Fig — The number of newly produced cells per hour was reduced in RNAi-AtCSN4 plants compared to Col-0 WT. The number of newly produced cells was determined by 72h period. The error bars represent standard errors. n = 10; *: p-value <0,05. (TIF) [file pgen.1007899.s006.tif]

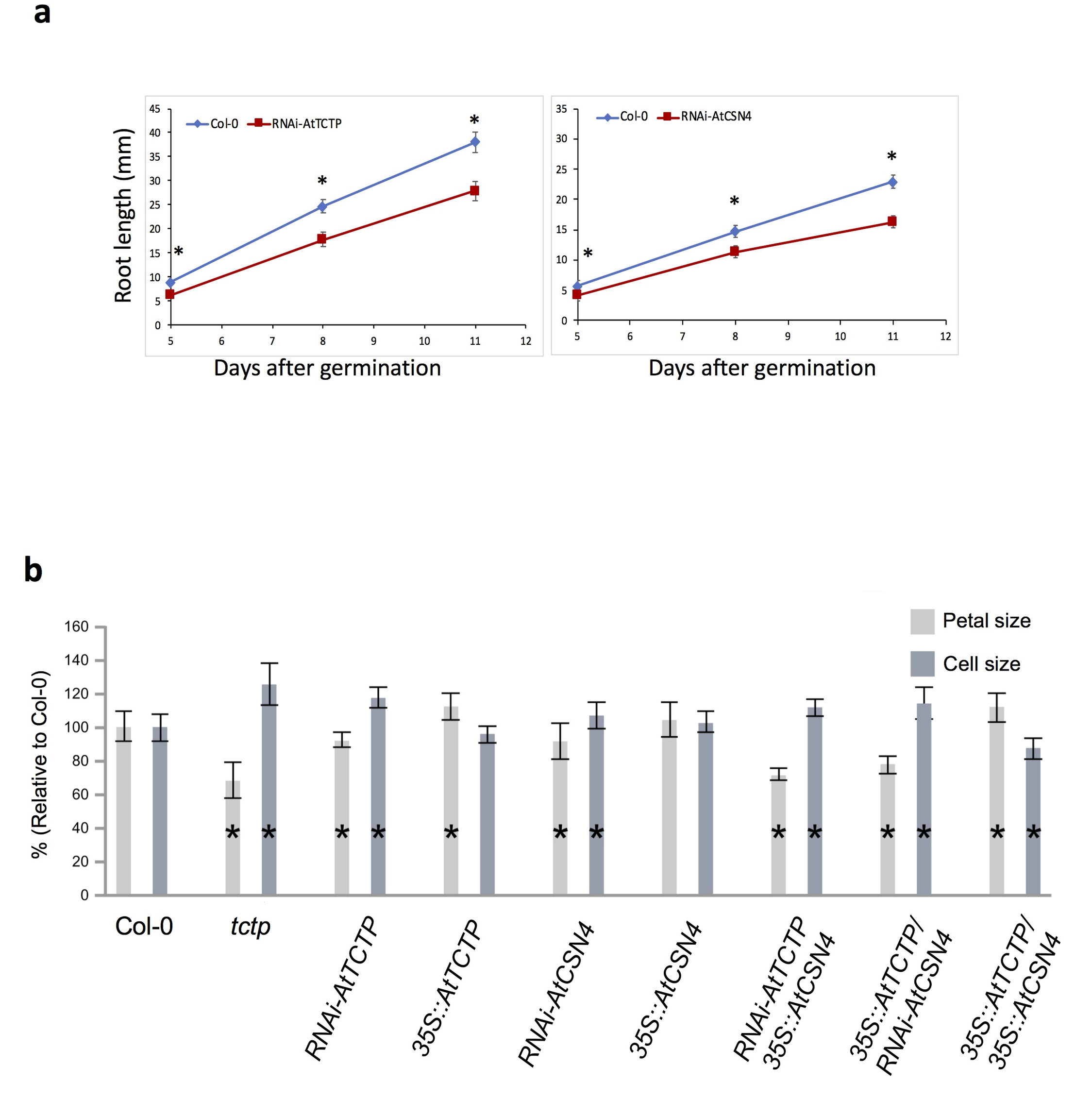

Supplement: S7 Fig — (a) RNAi-AtTCTP and RNAi-AtCSN4 plants exhibit reduced root growth compared to the wild-type (Col-0). Root length was measured at day 5, 8 and 11 days after germination. Values are average +/- standard error (n = 30 for RNAi-AtTCTP and n = 20 for RNAi-AtCSN4). Asterisks indicate statistically relevant differences (T-test; p-value <0.01). (b) Compared to the WT, mature petals of lines tctp, RNAi-AtTCTP, RNAi-AtCSN4, RNAi-AtTCTP/35S::AtCSN4 and 35S::TCTP/RNAi-AtCSN4 are reduced in size with increased cell size, suggesting lower cell division rate. Conversely, mature petals of lines overexpressing AtTCTP (lines 35S::AtTCTP) and the double overexpressor 35S::AtTCTP/35S::AtCSN4 are larger in size while cell size was unaffected or smaller, respectively, compared to Col-0. This suggest increased cell division rate in these lines. The stars indicate significant differences relative to the WT Col-0 (T-test; p-value < 0,001). (TIF) [file pgen.1007899.s007.tif]

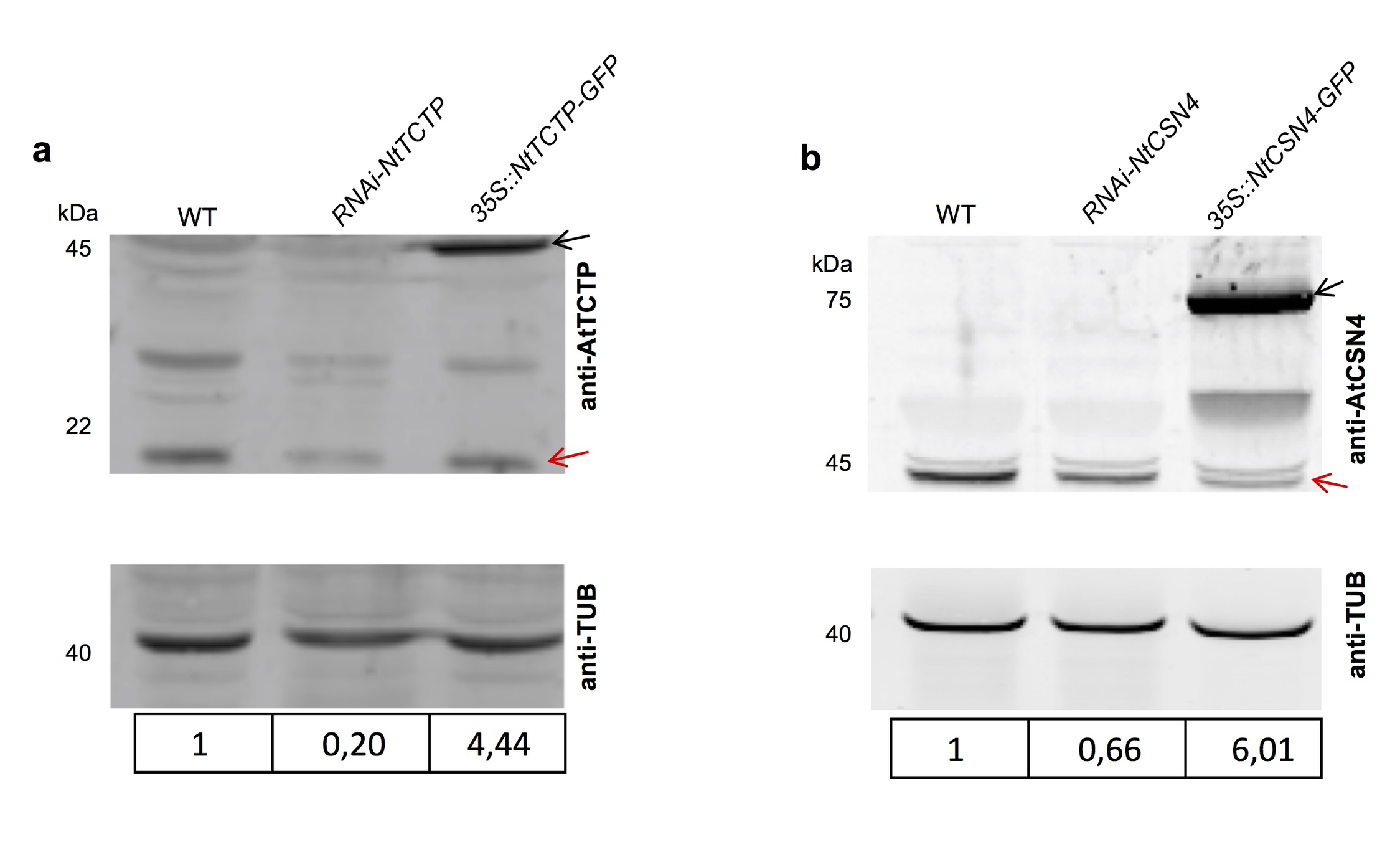

Supplement: S8 Fig — Western blot assay to evaluate the accumulation of NtTCTP (a) and NtCSN4 (b) in WT BY-2 tobbacco cells, and in BY-2 cells knockdown and overexpressor for these genes. The relative accumulation of NtTCTP and NtCSN4 based on Western blot data is shown under each lane. Black arrows indicate GFP fused proteins (NtTCTP-GFP or NtCSN4-GFP). Red arrows indicate endogenous NtTCTP and NtCSN4 proteins. (TIF) [file pgen.1007899.s008.tif]

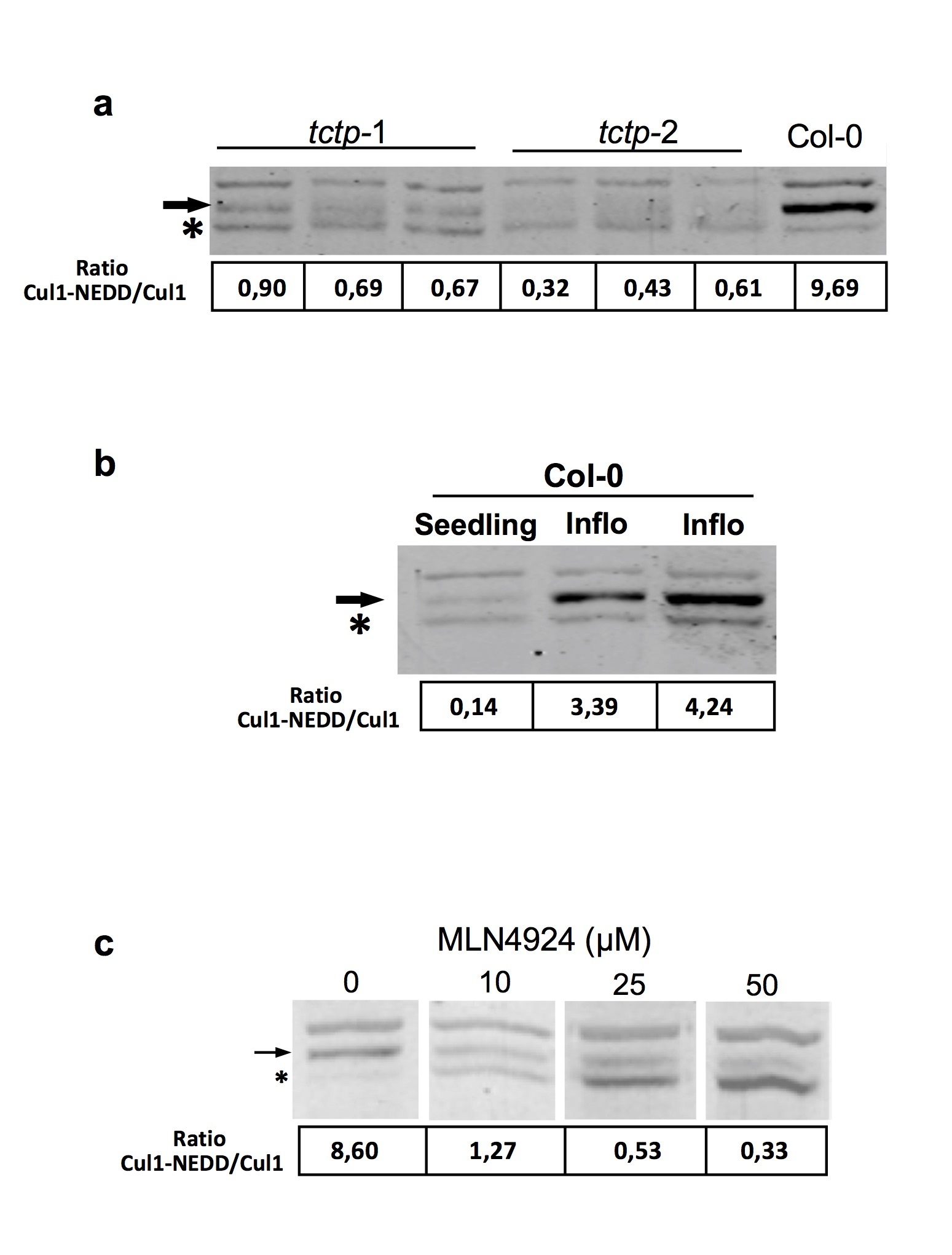

Supplement: S9 Fig — (a) CUL1 neddylation is decreased in tctp mutants. Three independent samples (1–3) were analyzed using two independent tctp knockouts (tctp-1 and tctp-2). (b) CUL1NEDD8/CUL1 ratio in inflorescence and seedlings of Col-0 plants. (c) Treatment with MLN4924, a drug that inhibits neddylation, results in an increase of the free CUL1 form with concomitant decrease of the CUL1NEDD8 form, confirming that the observed two bands correspond to neddylated and non neddylated CUL1. CUL1 protein was detected by Western blot using anti-CUL1 antibody. Quantification of CUL1NEDD8/CUL1 ratio is shown under each lane. Star: CUL1. Arrow: CUL1NEDD8. (TIF) [file pgen.1007899.s009.tif]

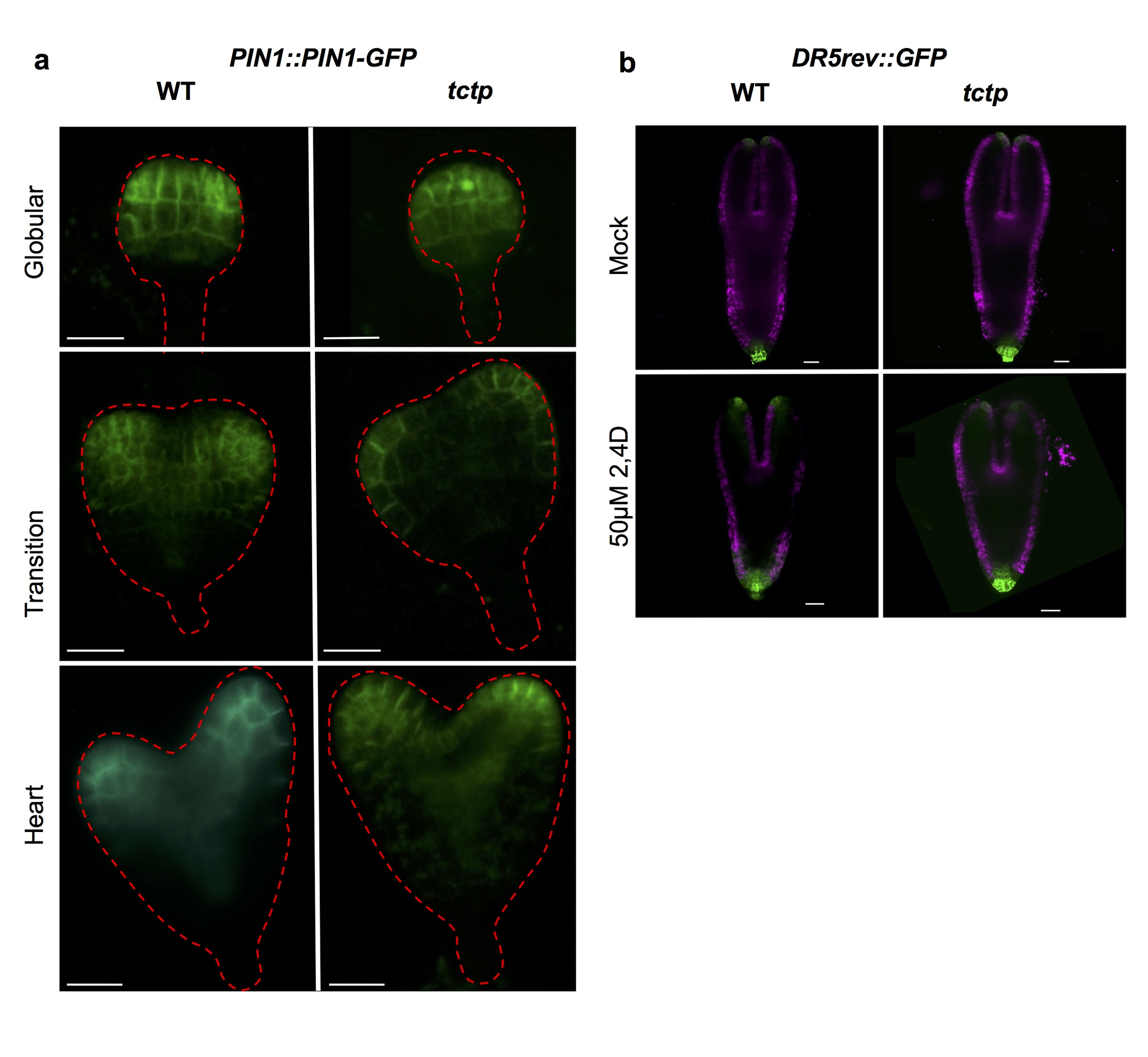

Supplement: S10 Fig — (a) PIN1::PIN1-GFP localization in tctp knockout embryos is similar to that in WT embryos, indicating that auxin efflux is not disturbed by tctp loss-of-function. Embryos at globular, transition and heart stages are shown. Bars: 2 0μm. (b) The accumulation of GFP, expressed under the control of synthetic auxin response DR5rev promoter, is not disturbed in tctp mutant embryos compared to WT embryos, indicating that auxin transduction pathway is not disturbed by tctp loss-of-function. Exogenous treatment with synthetic auxin, 2,4-D leads to similar expansion of DR5rev-GFP expression in tctp mutant and WT embryos. Bars = 20 μm. (TIF) [file pgen.1007899.s010.tif]
